# Identification and characterisation of novel CAR‐T cells to target IL13Rα2 positive human glioma in vitro and in vivo

**DOI:** 10.1002/ctm2.1664

**Published:** 2024-04-29

**Authors:** Pamela Leland, Heba Degheidy, Ashley Lea, Steven R. Bauer, Raj K. Puri, Bharat H. Joshi

**Affiliations:** ^1^ Tumor Vaccine and Biotechnology Branch Division of Cell Therapy II Silver Spring Maryland USA; ^2^ Cellular and Tissue Therapy Branch, Office of Cellular Therapy & Human Tissues, Office of Therapeutic Products Center for Biologics Evaluation and Research U.S. Food and Drug Administration, White Oak Silver Spring Maryland USA; ^3^ Wake Forest Institute for Regenerative Medicine Winston‐Salem North Carolina USA; ^4^ Iovance Biotherapeutics, Inc. Frederick Maryland USA

**Keywords:** CAR‐T cell therapy, genetically engineered T cells, IL‐13Rα2, immunotherapy, single‐chain variable fragment

## Abstract

**Background:**

Previously, we discovered that human solid tumours, but not normal human tissues, preferentially overexpress interleukin‐13Receptor alpha2, a high binding receptor for IL‐13. To develop novel anti‐cancer approaches, we constructed a chimeric antigen receptor construct using a high binding and codon optimised scFv‐IL‐13Rα2 fragment fused with CD3ζ and co‐stimulatory cytoplasmic domains of CD28 and 4‐1BB.

**Methods:**

We developed a scFv clone, designated 14‐1, by biopanning the bound scFv phages using huIL‐13Rα2Fc chimeric protein and compared its binding with our previously published clone 4‐1. We performed bioinformatic analyses for complementary determining regions (CDR) framework and residue analyses of the light and heavy chains. This construct was packaged with helper plasmids to produce CAR‐lentivirus and transduced human Jurkat T or activated T cells from peripheral blood mononuclear cells (PBMCs) to produce CAR‐T cells and tested for their quality attributes in vitro and in vivo. Serum enzymes including body weight from non‐tumour bearing mice were tested for assessing general toxicity of CAR‐T cells.

**Results:**

The binding of 14‐1 clone is to IL‐13Rα2Fc‐chimeric protein is ∼5 times higher than our previous clone 4‐1. The 14‐1‐CAR‐T cells grew exponentially in the presence of cytokines and maintained phenotype and biological attributes such as cell viability, potency, migration and T cell activation. Clone 14‐1 migrated to IL‐13Rα2Fc and cell free supernatants only from IL‐13Rα2+ve confluent glioma tumour cells in a chemotaxis assay. scFv‐IL‐13Rα2‐CAR‐T cells specifically killed IL‐13Rα2+ve but not IL‐13Rα2‐ve tumour cells in vitro and selectively caused significant release of IFN‐γ only from IL‐13Rα2+ve co‐cultures. These CAR‐T cells regressed IL‐13Rα2+ve glioma xenografts in vivo without any general toxicity. In contrast, the IL‐13Rα2 gene knocked‐down U251 and U87 xenografts failed to respond to the CAR‐T therapy.

**Conclusion:**

Taken together, we conclude that the novel scFv‐IL‐13Rα2 CAR‐T cell therapy may offer an effective therapeutic option after designing a careful pre‐clinical and clinical study.

## 1 INTRODUCTION

Chimeric antigen receptor (CAR) T cell therapy is a promising anti‐cancer immunotherapeutic strategy for hematological malignancies.^1^, ^2^, ^3^ Recent approvals of six CD19‐targeted CAR‐T cell products, Kymriah, Yescarta, Tecartus, Abecma, Breyanzi and Carvykti by the US FDA for treatment of B‐cell acute lymphoblastic leukemia (B‐ALL), diffuse large B‐cell lymphoma (DLBCL), mantle cell lymphoma, relapsed or refractory multiple myeloma, and relapsed or refractory large B‐cell lymphoma are considered milestones in the field, which has created enormous interest in the scientific community.^4^, ^5^ When CAR‐T cells bind the targeted tumour cells via antibody recognition, they trigger the CAR‐T cell cytolytic function, cytokine secretion, proliferation and killing of tumour cells.^6^, ^7^, ^8^, ^9^ Unfortunately, successful CAR‐T cell therapies are accompanied by serious adverse toxicities. The cytokine release syndrome (CRS) is a common serious complication observed in large percentage of subjects. Neurotoxicity is another serious adverse event seen in a subset of patients, which can be lethal.^10^, ^11^ Although CRS can be managed using anti‐IL‐6 antibody and steroids, the CAR‐T treatment warrants a careful monitoring of the subjects under medical supervision and management. The pathogenicity of these serious adverse events is not completely understood. It is believed that the cytokines and factors released by CAR‐T cells and dying tumour cells may generate these side effects.

IL‐13Rα2, a high affinity IL‐13 binding protein, is overexpressed in a number of solid human cancers such as glioblastoma multiforme (GBM), certain types of head and neck cancer, AIDS Kaposi's sarcoma, pancreatic cancer, prostate cancer, ovarian cancer and pulmonary cancer but with limited or no expression in normal tissues.^12^, ^13^ IL‐13Rα2 is overexpressed in ∼76% of GBM but is not detected in normal brain tissue or normal immune cells, making it an attractive target for the receptor‐directed cancer therapy.^14^, ^15^, ^16^, ^17^ It is also reported that IL‐13Rα2 gene belongs to the top 10% of 73 evaluated tumour‐associated antigens analysed by Nanostring digital RNA counting that were differentially expressed between tumours and normal tissues.^18^ While initial studies indicated that IL‐13Rα2 is a decoy receptor^19^, ^20^, ^21^, ^22^; more recent studies have demonstrated that IL‐13Rα2 prevents apoptosis and induces TGF‐β secretion.^23^, ^24^, ^25^ In addition, IL‐13Rα2 is associated with tumour progression and invasion leading to poor prognosis of subjects with IL‐13Rα2 overexpressing tumours.^26^, ^27^ Thus, immune targeting of IL‐13Rα2 as a tumour antigen provides a unique opportunity for a targeted immunotherapy for IL‐13Rα2 positive solid tumour.

In line with development of anti‐cancer therapeutic approaches to selectively target IL13Rα2 such as a chimeric fusion protein consisting of IL‐13 and truncated *Pseudomonas* exotoxin (IL‐13‐PE),^28^, ^29^ we generated a scFv for anti‐IL‐13Rα2 antibody by the phage display technology, which specifically recognised IL‐13Rα2 on tumour cell surfaces, with a high binding activity and specificity and fused with truncated *Pseudomonas* exotoxin (scFvIL‐13Rα2‐PE).^30^ In the current study, we report the development of a novel IL‐13Rα2‐specific CAR (IL‐13Rα2‐CAR‐T cells) with an improved scFv‐based antigen binding domain that provides better recognition of IL‐13Rα2 on the tumour cell surface. The scFv construct is fused with CD28 cytoplasmic, 4‐1BB and CD‐3ζ endodomains.^31^, ^32^, ^33^ Alteration in the intracellular domains has resulted in a different generation of CAR‐T cells from the first generation, which included a CD3ζ motif, to the second and third generations, which included one or two costimulatory domains, and new V_H_, variable heavy chain; V_L_, variable light chain regions. Newer generations of CAR‐T cell therapeutics have shown remarkable improvement and anti‐tumour activity in pre‐clinical settings, but these improved CAR‐T cells have not been reported in the clinic.^34^ These IL‐13Rα2‐CAR‐T cells can recognise and specifically kill only IL‐13Rα2‐positive and not IL‐13Rαl‐positive target cells in vitro via perforin/granzyme B pathway and in vivo animal models of human cancers. We have shown that IL‐13Rα2‐CAR‐T cells are biologically active and functional as they secrete IFN‐γ when exposed to IL‐13Rα2 positive tumour cells. Similarly, they are equally potent and specific in xenografts developed in NOD/Shi‐scid/IL‐2Rγnull (NOG) mice by regressing only receptor positive tumour cells.

## 2 MATERIALS AND METHODS

### 2.1 Cell lines

Human Jurkat‐T cells, U87MG and T98G glioma cell lines were obtained from ATCC and grown as per the supplier's instructions. The U251 glioma cell line was obtained from National Cancer Institute (NCI) and maintained in the Roswell Park Memorial Institute (RPMI) complete medium with 10% fetal bovine serum (FBS). T98G and U87MG glioma cell lines were maintained in the Eagle's Minimum Essential Medium (EMEM) complete medium supplemented with 10% FBS. We have previously characterised these cell lines for IL‐13Rα2 expression by RT‐PCR for mRNA and immunocytochemistry (ICC) analyses for protein expression as described by Joshi et al.^14^ Two IL‐13Rα2 positive glioma cell lines, U251 and U87MG were used for IL‐13Rα2 gene silencing by the siRNA technique using SureSilencing shRNA plasmids (cat# 336313 KH00597N, Qiagen, Gaithersburg, MD, USA) following supplier's instructions. These cell lines served as negative controls in some biological assays.

### 2.2 Design of lentiviral vector encoding scFv‐IL‐13Rα2‐CAR

A third‐generation CAR construct consisting of scFv antibody sequence against IL‐13Rα2 (as ectodomain), a CD28 transmembrane domain along with CD3ζ and CD28 and or 4‐1BB endodomains sequences was designed, codon‐optimised and synthesised by GenScript (GenScript, NJ, USA) in pUC57simple subcloning vector. The scFv clone #4 (control) was previously cloned in our laboratory using Griffith.1 library. These are applied phage display vectors used to make human antibodies from V‐gene repertoires from unimmunised donors. This is a large scFv library from the PBLs with greater than 10^7^ members made as diverse as possible by using both V_k_, and V_λ_, light chains, as well as V_HS_ derived from IgM and IgG mRNA.^35^, ^36^, ^37^ Phage particles expressing scFv were made from synthetic v‐gene segments and derived by cloning V_H_ and V_L_ human synthetic Fab Lox library vectors into phagemid vector pHEN2 as described previously.^30^ We utilised biopanning with repeat cycles of incubation, washing, amplification and reselection of scFv bound phage (clone 14‐1) by using Chinese Hamster Ovary (CHO) derived human IL‐13Rα2Fc (huIL‐13Rα2Fc) chimeric protein as antigen (cat# 7147‐IR, R&D Systems, Minneapolis, MN, USA) and codon optimising to develop CAR‐T construct, which was flanked by BamH1 and Not1 sites and placed into pCDH‐MSCV‐MCS‐EF1‐copGFP‐T2A‐Puro lentiviral vector (Cat# CD713B‐1, System Biosciences, Palo Alto, CA, USA). This transfer plasmid was packaged into 293T cells by co‐transfecting with three helper plasmids (pRRE, pRev and pCMV‐VSV‐G) to produce self‐inactivating (SIN) lentiviral vector expressing CAR‐IL13Rα2‐CAR, which was further purified by ultracentrifugation.

### 2.3 Relative binding activity of clone 4‐1 and 14‐1 by ELISA

For comparative assessment to analyse the binding activity of #14‐1 and #4‐1 scFv fragments, we performed enzyme‐linked immunosorbent assay (ELISA) based assay by coating immunoplates (96 wells) overnight at 4°C with 200 µL/well (4 µg/mL) with CHO‐derived recombinant huIL‐13Rα2Fc chimera. Plates were then washed three times with.05% Tween‐20 in PBS and blocked with PBS containing 2% milk. We compared the binding activities of previous scFv clone #4 as control and CHO‐derived scFv clone 14.1 in quadruplicate and the results were expressed as mean ± SD as described previously.^30^

### 2.4 Analysis of antigen binding residues and complementary determining regions in scFv IL‐13Rα2

To confirm the identity of scFv‐IL‐13Rα2, we performed standardised numbering methods to precisely define complementary determining region (CDR), frameworks and residues from the light and heavy chains that have an impact on the interaction and/or binding activity of the antibody for its target antigen. The ANARCI command line tool was employed to generate the numbering schemes. ANARCI facilitates the conversion of amino acid sequences into different numbering schemes.^38^ We analysed amino‐acid sequences using common numbering schemes of antibody variable domains to study the statistical variability in amino‐acid composition using the Kabat numbering scheme (https://www.ncbi.nlm.nih.gov/igblast). We also performed residue distribution at heavy and light chains and region sequence analysis.

We further analysed and compared these data with a structure‐based numbering scheme for antibody variable regions, which formed the CDRs and corrected the position numbers of the points within CDRL1 and CDRH1 per Chothia numbering scheme, Martin numbering scheme for correction of the insertion point within the framework region of heavy and light chains and ImMunoGeneTics (IMGT, The International ImMunoGeneTics information system, Montpellier, France) numbering scheme highlighting protein sequences of the immunoglobin superfamily including variable domains from antibody light and heavy chains as well as T cell receptor chains (http://www.imgt.org;https://github.com/oxpig/ANARCI,https://academic.oup.com/bioinformatics/article/32/2/298/1743894,http://www.abysis.org/abysis/sequence_input/key_annotation/key_annotation.cgi,http://www.imgt.org/3Dstructure‐DB/cgi/Collier‐de‐Perles.cgi andwww.chemogenomix.com).

### 2.5 Manufacturing of CAR‐T cells

Human PBMCs were isolated from buffy coats of normal healthy blood donors who donated blood at the Division of Transfusion Medicine, NIH. CAR‐T cells were generated from CD4 and CD8 +ve T cells isolated from normal human blood donor buffy coat using straight from buffy coat CD4 and CD8 microbead kit (Cat# 130‐114‐980 for CD4 microbead kit and Cat# 130‐114‐978 for CD8 microbead kit, Miltenyi Biotec, Waltham, MA, USA). For CD4/CD8 T cell activation, the cells were activated with anti‐CD3/CD28 antibody coated magnetic Dynabeads (cat# 11131D, ThermoFisher Scientific, Waltham, MA, USA) at a ratio of 3:1 (T cell to bead) and transduced with lentiviral vector at different multiplicity of infections (MOIs). T cells were maintained in culture at 1‐6 × 10^6^ cells/mL in the T cell culture medium (TCM) supplemented with 50 ng/mL IL‐2 (Cat# 130‐111‐160, Miltenyi Biotec, Waltham, MA, USA). These PBMC derived CAR‐T cells are termed as CAR‐T. A parallel set of experiments was set for transduction and expansion to confirm the identity of transgene and signalling domains in human Jurkat T cells (termed CAR‐Jurkat).

The identity of transduced T cells was confirmed by performing a combination of indirect immunofluorescence assay for transgene and fluorescence activated cell sorting (FACS) based assays for CD28 and CD3ζ domains as described below because of non‐availability of transgene detecting antibody (Lea A, et al., unpublished observations).

Indirect immunofluorescence assay for transgene identification in transduced CAR‐Jurkat and CAR‐T cells:

For detection of scFv‐IL‐13Rα2 expression on transduced Jurkat or T cells, we developed an indirect immunofluorescence assay and compared its performance with FACS as described previously.^39^ Briefly, we plated either 75 000 Jurkat‐CAR or CAR‐T cells in a poly‐L‐Lysine coated 4‐well chamber slide. We biotinylated recombinant human IL‐13Rα2Fc chimeric protein (Cat# 7147‐IR, Biotechne, R&D systems, Minneapolis, MN, USA) by using EZ‐Link micro sulfo‐NHS‐Biotinylation kit (Cat# 21925, Thermo Scientific, Waltham, MA, USA). Transduced Jurkat and T cells were then incubated with 500 ng/mL purified biotinylated recombinant human IL‐13Rα2Fc chimera protein followed by streptavidin‐Alexa 594 (.5 µg/mL) to develop red fluorescence in scFv‐IL‐13Rα2 CAR‐expressing cells. The cells expressing ≥ 2+ fluorescence intensity were counted at 200× magnification by viewing in NIKON epifluorescence microscope. Each value is mean ± SD of quadruple experiments determined in a blinded manner by three independent investigators and data expressed as % positive cells.

### 2.6 Flow‐cytometry analysis

BD FACSCanto or FACSCalibur (BD Bioscience, San Jose, CA, USA) instruments were used to acquire immunofluorescence data which were analysed with CellQuest (BD Bioscience) or FlowJo v.7 (FlowJo, LLC Ashland, OR, USA) software for final data analysis and graphic representation. Isotype control was immunoglobulin IgGl‐PE (Cat# 68995, mouse (G3A1) mAb IgG1 isotype control‐PE, Cell Signaling Technology, Denver, MA, USA). CD28 cytoplasmic and CD3ζ endodomains were immunostained with anti‐CD28.2 mouse mAb PE conjugate (Cat# MA1‐10170, Thermofisher Scientific, Carlsbad, CA, USA) or CD3ζ monoclonal antibody PE conjugate (Cat# 12‐2479‐82, CD247 monoclonal antibody (6B10.2) PE, eBioscience‐ThermoFisher Scientific, Carlsbad, CA, USA) after permeabilising CAR‐Jurkat or CAR‐T cells. Similarly, both CAR‐Jurkat and CAR‐T cells were immunostained with anti‐human 4‐1BB FITC antibody (cat# 11‐1379‐42, eBioscience‐ThermoFisher Scientific, Carlsbad, CA, USA) for FACS analyses. We permeabilised CAR‐Jurkat and CAR‐T cells for detecting CD28 and CD3ζ endodomains by using a commercial reagent (Cat#954714, BD Biosciences, Franklin Lakes, NJ, USA) before immunostaining with respective antibodies in the FACS assay. The data were expressed as number of cells counted in normalised to mode values. Each datum is a mean ± SD of three independent experiments performed in triplicate.

### 2.7 Cell viability and proliferation analysis of CAR‐T cells

The cell viability of CAR‐T cell cultures was examined during cell expansion by the trypan blue exclusion technique. To further assess the health of CAR‐T cells, cellular proliferation was assessed by the CellTiter 96R AQuesous one solution kit (MTS assay; Cat# G3582, Promega, Madison, WI, USA). Following manufacturer's instructions, the experiments were performed in quadruplicate and the results were expressed as mean ± SD.

### 2.8 Migration of CAR‐T cells in vitro

We measured migration potential of CAR‐T cells in 24‐well ChemoTx plates with a 5‐µm pore diameter (cat# ab235693, abcam, Cambridge, MA, USA) (Chemotaxis assay) as described previously.^39^ Percentage migration was calculated as the number of cells in the lower chamber divided by the total number of cells plated per well. Each value is expressed as mean ± SD of four independent experiments.

### 2.9 Cytotoxic activity

For measuring cytotoxic activity of CAR‐T cells in vitro, we tested IL‐13Rα2 positive and IL‐13α2 knock‐down (KD) U251 and U87MG glioma cell lines as described previously.^39^ Briefly, these tumour cells were labelled by intracellular Calcein violet‐acetoxymethyl ester and release of calcein violet in the supernatants was recovered at the end of 6 h of co‐culture of target: effector cells in the ratio of 1:10, 1:20, 1:30, 1:40 and 1:50. Fluorescence is measured quantitatively on a fluorescent plate reader (Spectramax M5 at Ex/Em = 400/452 nm). The data are shown as mean ± SD of three independent experiments performed in quadruplicate involving co‐cultures of tumour and CAR‐T cells.

In addition, the IL‐13Rα2 gene was silenced by the SureSilencing shRNA Plasmids technique using the siRNA sequence GCTACCATTTGGTTTCATCTT for transfection of U251 and U87 MG cell lines as per manufacturer's protocol (cat# 336313 KH00597N, QIAGEN, Germantown, MD, USA) as described previously.^39^ We maintained the gene‐silenced cell lines in the complete medium for 12 passages (∼50 days). We harvested 10^7^ cells for RNA extraction using RNA plus mini extraction kit (Cat# 74134, Qiagen, Germantown, MD, USA) at specified time‐points, RT‐*q*PCR was performed for IL‐13Rα2 RNA expression in gene silenced glioma cell lines as described previously.^14^, ^40^ The data are shown as mean ± SD of four independent experiments performed in quadruplicate involving co‐cultures of target and effector CAR‐T cells.

### 2.10 IFN‐γ release

IL13Rα2‐CAR‐T cells (100 000) were co‐cultured for 20 h with an equal number of IL‐13Rα2 positive, IL‐13Rα2 negative or IL‐13Rα2 KD tumour cells in the 96‐well round bottom plate. The cultures were centrifuged at 3500×*g* for 10 min at the end of the incubation period and supernatants were harvested for quantitative determination of IFN‐γ secretion by ELISA assay (Cat # 430115, Bio legend, San Diego, CA, USA).

### 2.11 Functional titer of scFV‐IL‐13Rα2‐CAR‐GFP vector by FACS

For FACS titration of the lentivirus, we used lentivirus with copGFP as reporter gene because of the ease of measuring its fluorescence by FACS analysis. The experiment was performed on HEK293T cells in a 6‐well plate (80 000 cells/well). At 24 h post‐seeding, cells from one well were counted (to determine the number of transducing cells) and transduced with serial 10‐fold dilutions of lentivirus: undiluted, 1/10 and 1/100. After 72 h, the cells were detached from the plate (using trypsin–ethylenediaminetetraacetate (EDTA)) and the lentiviral biological titer was estimated by FACS‐based CopGFP fluorescence.

### 2.12 Endotoxin levels

Endotoxin levels of final CAR‐T cell products were determined using a chromogenic ELISA‐based assay kit (Cat# A39552, ThermoFisher Scientific, Waltham, MA, USA) following manufacturer's instructions and absorbance was measured at 405 nm by utilising a SpectraMax M5 plate reader. Test samples from four lots of CAR‐T cell batches derived from normal healthy blood bank donors were tested in quadruplicate and values were calculated as mean ± SD.

### 2.13 Viral copy number determination

We measure the viral copy number of lentiviruses stably integrated into the transduced CAR‐T cell DNA. The DNA‐qPCR method for lentiviral titration analysis was optimised and performed following the protocol as described by Barczak et al.^41^ For VCN enumeration, we used Woodchuck Hepatitis Virus Posttranscriptional Regulatory Element (WPRE) sequence‐based primers. Total genomic DNA was extracted from five engineered CAR‐T cell product batches using a mini column‐based DNA isolation kit (Cat# ID 69504, Qiagen, QIAGEN, Germantown, MD, USA) and amplified using SsoAdvanced Universal SYBR Green supermix (Cat# 1725271, Bio‐Rad, Hercules, CA, USA) on a CFX96 touch‐real‐time PCR detection system (Bio‐Rad, Hercules, CA, USA). Plasmid pLVX‐TetOne‐Puro vector contains the WPRE element (cat# 631847, Takara Bio USA, San Jose, CA, USA), so was used as positive control in the assay to generate a standard curve of known concentrations of plasmid lentiviral DNA (1 × 10^4^ to 1 × 10^9^ copies/reaction). Primer sequence used for VCN determination in the assay:

WPRE Forward: 5′‐GTCCTTTCCATGGCTGCTC‐3′

WPRE Reverse: 5′‐CCGAAGGGACGTAGCAGA‐3′

The DNA from CAR‐T cells manufactured from five buffycoat samples was analysed for amplification in four replicates. The number of copies/CAR‐T positive cell was calculated as suggested^41^ and data were calculated are expressed as mean ± SD.

### 2.14 Tumour xenograft studies

U251 and U87 human glioma cells (5 × 10^6^) were injected subcutaneously in the right flank of Taconic mice (female, 4−6 weeks old, NOD). Cg‐Prkdcscid IL2rgtm 1Sug/Jic Tac mice (NOG; Taconic Bioscience Inc.) were used in all of the experiments. The mice were maintained under pathogen‐free conditions under protocols approved by the institutional guidelines and approval by local authorities. All animal studies were reviewed and approved by the Center for Biologics Evaluation and Research, FDA, Animal Care and Use Committee (IUCAC Protocol 2003−08). Infusion of untransduced T cells by tail vein served as controls in the study. Tumour progression and size were monitored by measuring horizontal and vertical diameters by vernier caliper. After tumours were palpable (5 × 4 mm in diameter in ∼ 10−12 days), mice were treated with 5 and 10 × 10^6^ CAR‐T cells. Additionally, we included U251 KD and U87MG KD glioma cells for the xenograft development in mice as control and treated with same two doses of CAR‐T cells. Tumour growth and their general health were also monitored in these mice. The mice were randomised and blindly followed up for next 8 weeks for tumour growth. When tumours reached 200 mm^2^, they were humanely euthanised by CO_2_ asphyxiation according to Institutional Animal Care and Use Committee (IACUC) protocol.

Approximately, 100 mg of tumours were homogenised in RNA lysis buffer and RNA was extracted using RNAesay plus mini kit (Cat# 74134, Qiagen, Germantown, MD) to determine IL‐13Rα2 levels at the end of the experiments. All surviving mice were euthanised using same CO_2_ asphyxiation protocol at the end of experiments.

### 2.15 General toxicity and serum chemistry

For general toxicity due to the CAR‐T cell therapy in mice, we included a group of non‐tumour bearing mice and administered two doses of CAR‐T cells. We measured the body weight of all mice once a week for 90 days. We collected the serum before CAR‐T administration and at the end of the experiment before euthanisation of mice. Serum enzymes such aldolase, alanine aminotransferase (ALT) (cat#, Sigma‐Aldrich, St. Louis, MO, USA), creatine kinase (CK) activities and serum creatinine values (cat# ab65340 and 15590, abcam, Boston, MA, USA) were determined after following manufacturer's protocol. Each parameter was assayed in triplicate and each value is expressed as mean ± SD.

### 2.16 Statistical analysis

The data were compared using unpaired Student's *t*‐test analyses. *P*‐values < .05, calculated by using GraphPad Prism software (Graph‐ Pad Software, La Jolla, CA, USA) were considered to determine a significant difference. Two‐way analysis of variance was used to compare labelling conditions (*n* = 4) and the Wilcoxon test was used to obtain two‐sided global *P*‐values for cell survival or proliferation (*n* = 4). CAR‐T treated mice were followed up for 90 days for monitoring survival time. Survival curves were generated by the Kaplan–Meier method using SAS software (SAS Institute Inc., 2023, Version 9.4) and compared by using the log‐rank test. In addition, adjustment for multiple comparisons for the log‐rank test was performed for treated and control group of experimental mice to evaluate the data for strata comparison.

## 3 RESULTS

### 3.1 Characterisation of scFv‐IL‐13Rα2 CAR construct

We developed a lentiviral vector encoding scFv derived from anti‐IL13Rα2 antibody identified from a phage display library schematically shown in Figure S1. As shown in Figure S1B, the binding activity of scFv clone 14‐1(new clone) was ∼5 times better in binding with CHO derived recombinant huIL‐13Rα2Fc chimeric protein (*P* ≤.001), compared with previously developed clone #4 as control using recombinant huIL‐13Rα2Fc protein coated ELISA plates suggesting that the clone 14‐1 (newer scFv fragment) was superior in binding with IL‐13Rα2Fc antigen. Amino acid sequence alignment data of #4 and #14‐1 showed that the CDRH3 region of scFv clone #14‐1 differed at position 102 through 104 HMI resides compared to RQS in clone #4. Similarly, the CDRL3 region showed the presence of M residue in #14‐1 instead of G residue at position 225 residue (Figure S1C).

**FIGURE 1:**
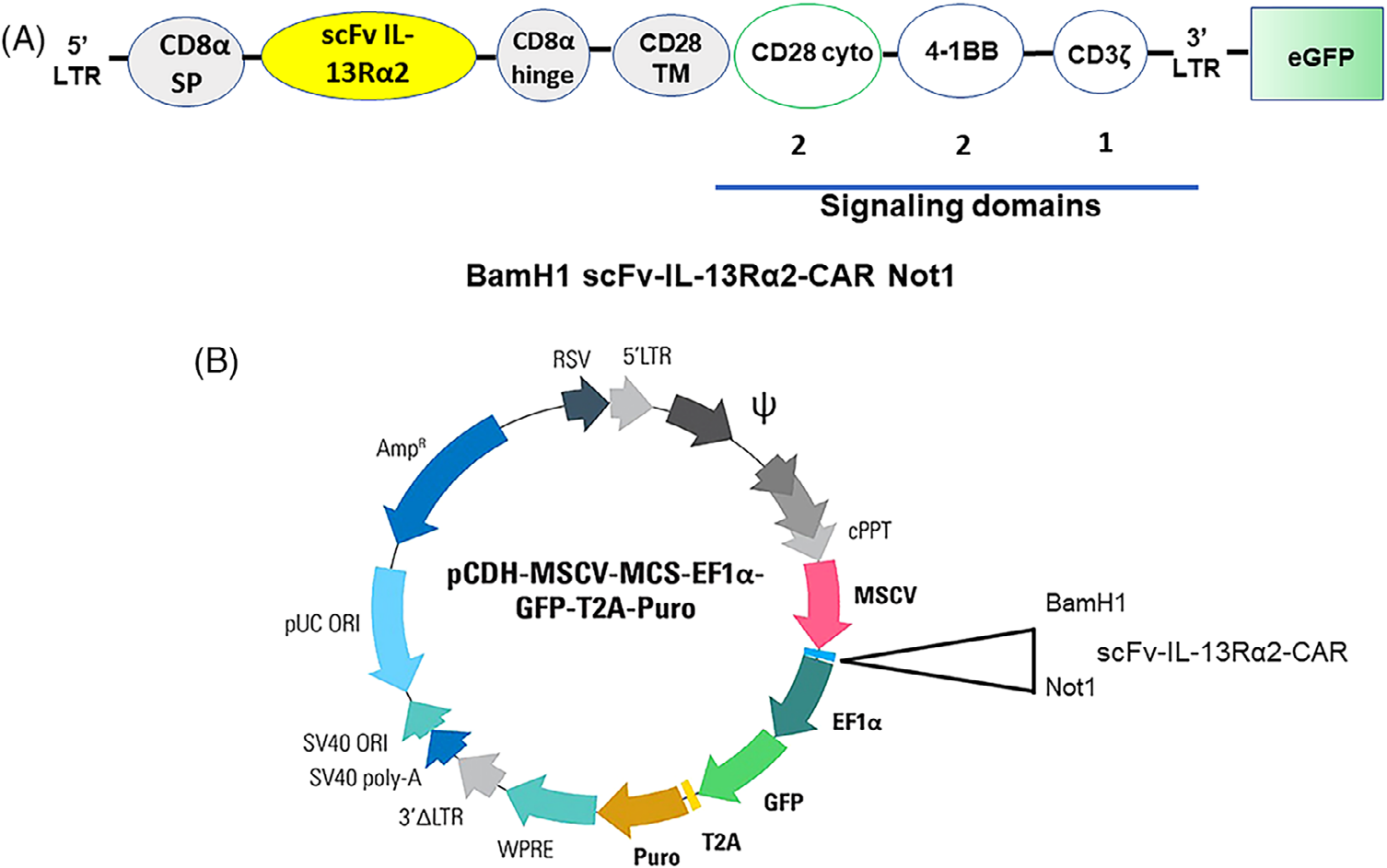
(A) Human scFv‐IL‐13Rα2 CAR construct: schematic diagram of scFv‐IL‐13Rα2 CAR construct. The construct contained a 5′‐CD8a signal peptide, codon optimised gene for scFv IL‐13Rα2, CD8a hinge, CD28 transmembrane and signal endodomains from CD28 cytoplasmic domain, 4‐1BB and CD3ζ. (B) Schematic diagram of lentiviral vector: the entire construct flanks between BamH1 (5′) and Not1(3′) and placed in pCDH‐MSCV‐MCS‐EF1α‐GFP‐T2A‐Puro lenti vector.

The construct contained an N‐terminal CD8α leader sequence, a codon optimised synthetic gene encoding anti‐IL‐13Rα2 scFv, a CD8α hinge region, a CD28 transmembrane domain, CD28 cytoplasmic domain, 4‐1BB co‐stimulatory endodomains, and CD3ζ domain placed in frame in lentiviral vector pCDH‐MSCV‐MCS‐Ef1a‐GFP‐T2A‐Puro (Figure 1A,B).

### 3.2 Identity and functional characterisation of scFv‐IL‐13Rα2 CAR construct

Next, we analysed the CAR‐T construct by DNA sequence analysis including flanking regions in a lentivirus vector between BamH1 and Not1 restriction sites within multiclonal sites of lentivector pCDH‐MSCV‐MCS‐Ef1‐GFP‐T2A. As shown in Figure S2A, the sequencing data confirmed that the CAR construct is 1631 bp long and contains 60.35% GC. It consists of sequences of CD8 signal peptide, scFv‐IL‐13Rα2 transgene, CD8 hinge, CD28 transmembrane domain, CD28 cytoplasmic domain, 4‐1BB, and CD3ζ domains. Its translated amino‐acid sequence is 540 residues long as shown in Figure S2B. The position of different domains of amino‐acid and their gene accession number are provided in Tables 1 and 2.

**TABLE 1:** Start and stop position of different domains in the scFv‐IL‐13Rα2 CAR construct.

Domain	Amino‐acid position
BamH1	1–2
**CD8a sig peptide**	**3**–**23**
**scFv‐IL‐13Ra2**	**24**–**264**
**CD8a Hinge**	**265**–**310**
**CD28 TM**	**311**–**340**
**CD28 cyto**	**341**–**380**
SalI restriction site	381–382
**4‐1BB cyto**	**383**–**424**
Three Glycine linker	425–427
**CD3 zeta**	**428**–**540**

**TABLE 2:** Gene accession number and position of different amino‐acids selected for constructing scFv‐IL‐13Rα2 CAR construct.

Domain	Gene accession number	Amino‐acid position
CD8a sig peptide	NM_001768	1–21
scFv‐IL‐13Ra2	Transgene	Our invention
CD8a Hinge	NM_001768	135–181
CD28 TM	BC093698	153–180
CD28 cyto	BC093698	181–220
4‐1BB cyto	NM_1561	214–255
CD3 zeta	BC025703	52–164

### 3.3 Identification of scFv‐IL‐13Rα2 transgene in transduced Jurkat and T cells

**FIGURE 2:**
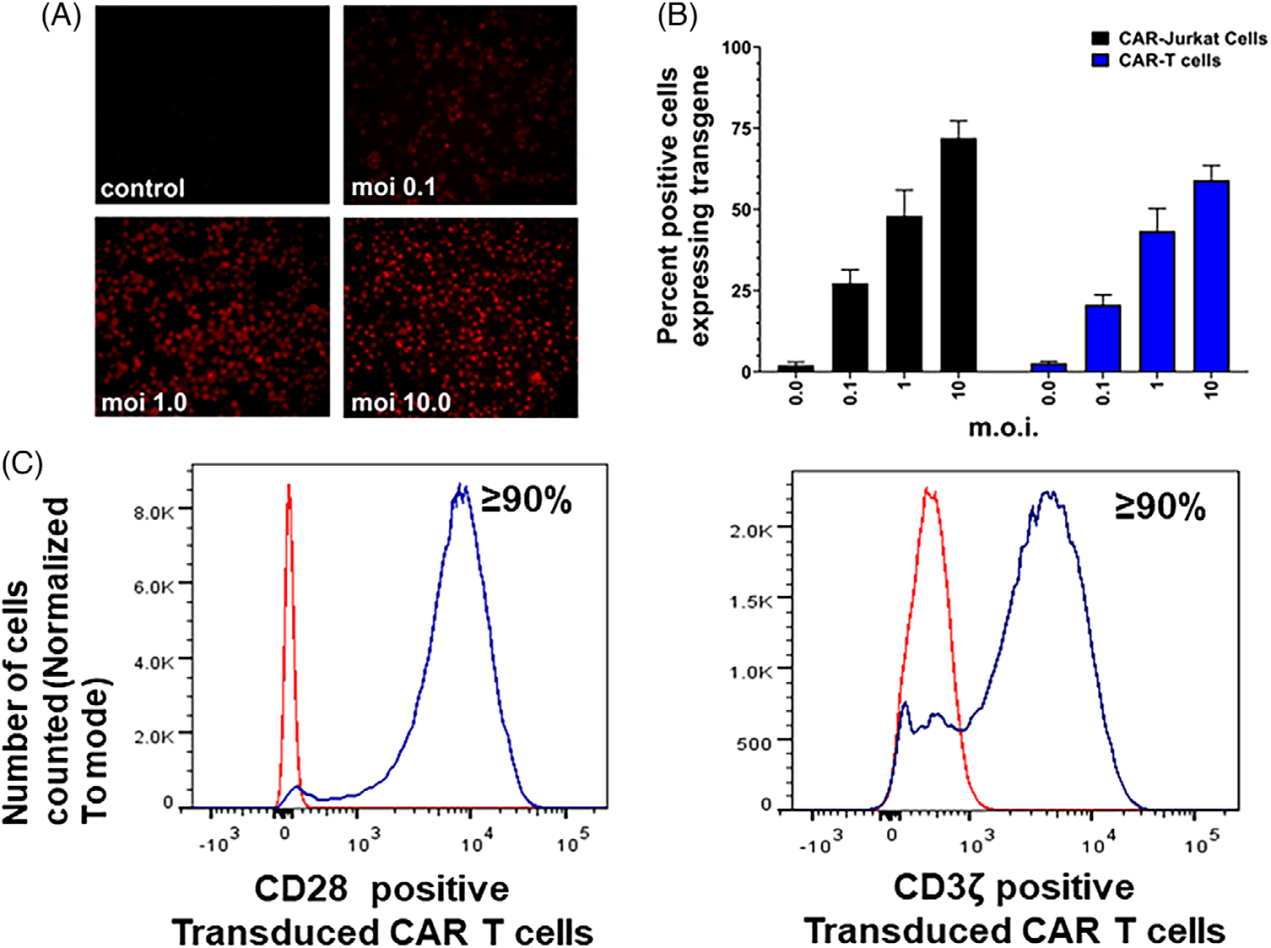
(A) Transduced CAR‐T cells were incubated with 500 ng/mL biotinylated recombinant human IL‐13Rα2 Fc chimeric protein followed by streptavidin‐alexa 594 to develop red fluoresce in scFv‐IL‐13Rα2 expressing cells. (B) The cells expressing ≥ 2+ fluorescence intensity were counted at 200× magnification by viewing with a NIKON epifluorescence microscope. Each value is mean ± SD of quadruple experiments determined in a blinded manner and expressed as % positive cells. (C) FACS analysis of transduced CAR‐T cells stained with anti‐CD28 and CD3ζ antibodies.  Histograms show the overlay of CD28 and CD3ζ positive cells with the corresponding control. The data were expressed as normalised to mode values.

Immunofluorescence analysis (IFA) for the detection of scFv‐IL‐13Rα2 transgene in transduced Jurkat and CAR‐T cells revealed an m.o.i.‐dependent increase in transgene positive cells. CAR‐Jurkat cells expressed slightly higher transgene levels compared to CAR‐T cells (Figure 2A,B). We performed FACS analysis to identify CD28 cytoplasmic domain and CD3ζ domain in transduced CAR‐T cells after permeabilisation. As shown in Figure 2C, in three independent experiments, more than 90% of CAR‐T cells expressed both CD28 and CD3ζ genes after transduction with 5 m.o.i. of scFv‐IL‐13Rα2 CAR construct. Similar results were observed for 4‐1BB domain expression in CAR‐Jurkat and CAR‐T cells (data not shown).

### 3.4 Endotoxin levels and viral copy number in CAR‐T + cells

Endotoxin levels from five engineered CAR‐T + cell product batches were measured by colorimetric assay as described in Section 2. The chromogenic assay for endotoxin could detect the endotoxin levels rapidly in the final CAR‐T cell product derived from five buffycoat samples from normal healthy blood donors. The data shown in Table 3 revealed that the mean endotoxin values ranged between 2.8 ± .21 and 4.6 ± .18 EU/mL.

**TABLE 3:** Evaluation of endotoxin levels in scFv‐IL‐13Rα2 CAR‐T cell product runs by chromogenic endotoxin quant assay.

Number of run	MOI	Mean endotoxin levels (EU/mL)*
Run 1	5	2.8 ± .21
Run 2	5	3.6 ± .28
Run 3	5	3.2 ± .11
Run 4	5	4.6 ± .18
Run 5	5	3.4 ± .24

* Each value is mean ± SD of four replicate values obtained after performing five independent experiments utilising Chromogenic Endotoxin Quant kit.

As shown in Table 4, the viral copy number analysis was performed by real‐time PCR based assay from genomic DNA extracted from five normal healthy donor derived CAR‐T cell products as described in Section 2. The mean values/CAR‐T cells ranged from 2.6 ± .19 −3.1 ± .20. Four replicate samples were tested and each value was mean ± SD of each individual product lot.

**TABLE 4:** Determination of vector copy number of scFv‐IL‐13Rα2 CAR‐T cell product runs by qPCR for WPRE gene.

Manufacturing	MOI	Mean copies/cell	mean copies/ CAR+ T cell*
Campaign			
1	5	1.8 ± .21	2.7 ± .17
2	5	2.6 ± .28	3.2 ± .22
3	5	2.2 ± .11	2.6 ± .19
4	5	1.6 ± .18	2.5 ± .12
5	5	2.4 ± .24	3.1 ± .20

* Each campaign used four buffycoat samples to manufacture the individual CAR‐T drug product. Four replicate samples of five independent experiments were tested and each value represents mean ± SD of each campaign.

### 3.5 Analysis of antibody variable domains of scFv‐IL‐13Rα2 by residue numbering schemes

We analysed the variable domain of IL‐13Rα2 by the residue numbering scheme to define CDRs, frame works and amino‐acid residues from heavy and light chains, which can influence binding activity and specificity. These data revealed eight different regions with varying number of residues and length for the heavy chain (Figure S3A,B). The sequences exhibited variable lengths of gaps where insertions could only be included at precise positions. Our data analysis revealed that the amino acid residues 27−38 in ascending loop B (CDR1), 56−59 in ascending C loop, 62−65 in descending C loop C (CDR2), 105−110 in ascending loop F and 113−117 in descending loop F (CDR3) are structural constituents (Figure S3C) of the heavy chain.

Our sequence analysis for the light chain showed nine different regions including a tail region of 275 amino acids (Figure S4A,B). Similar to heavy chain structural analysis for amino acid residues, we observed that in light chain amino acids 24−29 in the ascending B loop and 36−39 in the descending C loop belong to CDR1‐light, 56−57 in the ascending C loop and 65−69 in the descending C loop to CDR2‐light and 105−108 in the ascending F loop and 114−117 descending G loop to CDR3‐light, which are conserved (Figure S4C).

The distribution of amino acids in the heavy chain and the light chain is shown in Figure S5A,B after analysing them with the Kabat numbering scheme. We also analysed Kabat numbering of scFv residue sequence for a comparative alignment analysis with IMGT, Chothia and Martin numbering schemes for antibody variable regions, loop structures that formed the CDRs, position numbers of the insertion points within CDR‐H1 (heavy) and CDR‐L (light) chains, including variable domains from the antibody heavy chain (Figure S6A) and light chain (Figure S6B) and amino acid sequence alignment of the germ‐line V. These analyses revealed a structural position of amino‐acids that are involved in antigen binding and displaying hypervariable amino‐acid composition.

### 3.6 Viability and proliferation of CAR‐T cells

**FIGURE 3:**
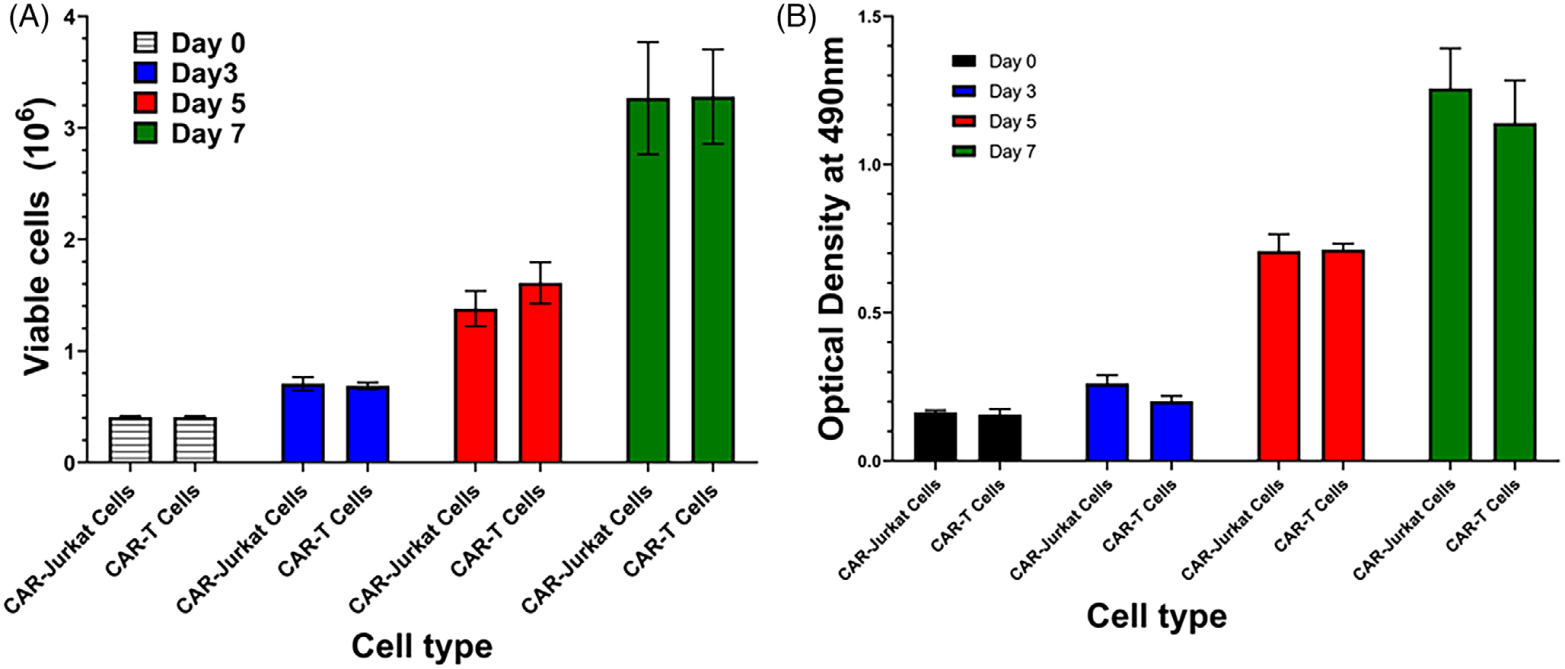
Analysis of biological quality attribute of CAR‐T cells. (A) Cell proliferation: proliferation of CAR‐Jurkat and CAR‐T cells at the indicated days of culture was determined by trypan blue exclusion technique and expressed as total number of viable cells. (B) Metabolic activity of CAR‐T cells is assessed by measuring optical density of reduced MTS tetrazolium by proliferating CAR‐T cells at 490 nm on specified time points. Each value is a mean ± SD of four independent experiments.

We determined the cell viability and cell proliferation of CAR‐Jurkat and CAR‐T cells up to 7 days of culture. As shown in Figure 3A, both CAR‐Jurkat and CAR‐T cells continued to grow for 7 days and maintained their cell viability. Similarly, both CAR‐Jurkat and CAR‐T maintained their metabolic and proliferative activity for 7 days as determined by MTS assay (Figure 3B).

### 3.7 Cytotoxic activity, potency and cell migration of CAR‐T cells

**FIGURE 4:**
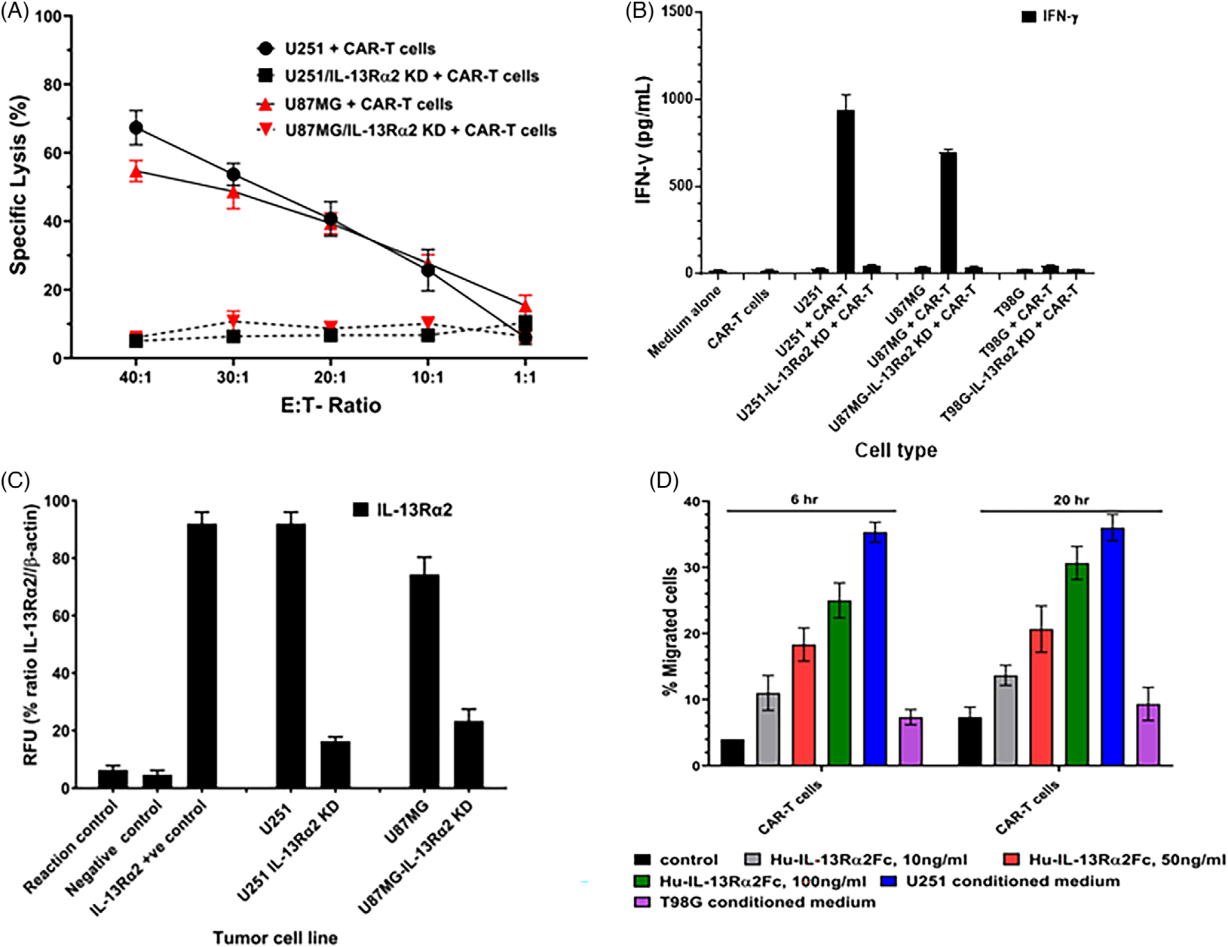
Biological functional properties of CAR‐T cells. (A) CAR‐T cells killed IL‐13Rα2 expressing tumour cell lines in a cell number dependent manner after co‐culturing a fixed number of Calcein‐violet loaded tumour cells (5000) as target cells. Gene knock‐down (KD) tumour cells served as negative control. (B) Co‐culture of CAR‐T effector cells with IL‐13Rα2 positive U251, and U87MG target cells released IFN‐γ in 20‐h incubation time. Cell free supernatants from IL‐13Rα2 negative T98G glioma cells or IL‐13Rα2 KO U251/U87MG showed basal amounts of IFN‐γ. (C) Total RNA from U251, U87MG and T98G glioma cells were reverse transcribed, and qPCR was performed using Il‐13Rα2 primers as described in Section 2. Results were expressed as % ratio of relative fluorescence units of IL‐13Rα2/ b‐actin gene. Each value is a mean ± SD of four independent experiments performed in triplicate. (D) Cell migration and invasion ability of scFv‐IL‐13Rα2‐CAR‐T cells was studied at 6 h and 20 h time points in Boyden chamber cell migration assay in response to different concentrations of rIL‐13Rα2Fc chimeric protein. No such migration was observed in control or conditioned medium from IL‐13Rα2 negative T98G tumour cell cultures. Each value is a mean ± SD of four independent experiments performed in triplicate.

The potency of CAR‐T cells was first assessed by cell killing assay against IL‐13Rα2 positive U251 and U87MG malignant glioma tumour cells lines, as these tumour cell lines express high levels of IL‐13Rα2 (Figure 4A). CAR‐T cells caused cell killing of two tumour cell lines (U251 and U87MG) in an effector cell number dependent manner (*P* ≤ .01). The killing of tumour cells was highly specific to the IL‐13Rα2 expression on target tumour cells as gene KD by gene silencing of IL‐13Rα2 on target tumour cells significantly eliminated cytotoxic activity of CAR‐T cells. Corresponding controls of IL‐13Rα2 KD U251 and U87MG clones were developed by the siRNA technology in which more than 90% IL‐13Rα2 gene was knocked down (Figure 4C) and sustained for more than 35 days (Figure S7A).

We next determined the potency of CAR‐T cells by IFN‐γ release assay when CAR‐T effector cells were co‐cultured with two different IL‐13Rα2 positive target glioma tumour cell lines (U251 and U87MG) and one IL‐13Rα2 negative glioma cell line (T98G). CAR‐T cells were co‐cultured with equal numbers of target cells for 20 h and IFN‐γ secretion was measured from the supernatant by ELISA. As shown in Figure 4B, the CAR‐T cells produced large and approximately equal amount of IFN‐γ in the supernatant when cultured with IL‐13Rα2 positive glioma cell lines. In sharp contrast, the CAR‐T cells made basal and minimal amount to IFN‐γ when cultured with IL‐13Rα2 negative or IL‐13Rα2 KD tumour cell line.

Next, we examined the cell migration and invasion ability of CAR‐T cells in a ChemoTx assay. CAR‐T cells were exposed to three different concentrations of recombinant IL‐13Rα2Fc chimeric protein or conditioned medium obtained from IL‐13Rα2 positive (U251) and IL‐13Rα2 negative (T98G) human glioma cell lines. As shown in Figure 4D, CAR‐T cell cultures from day 8 of the expansion phase invaded and migrated to human IL‐13Rα2Fc in a concentration‐dependent manner at 6 and 20 h time points. Similarly, CAR‐T cells invaded and migrated to the conditioned medium from IL‐13Rα2 positive U251 and U87 glioma cells but not to the conditioned medium from IL‐13Rα2 negative T98G glioma cells.

### 3.8 Sensitivity of IL‐13Rα2 positive glioma xenografts to scFv CAR‐T cell in vivo

In the present study, we observed a significant level of IL‐13Rα2 gene silencing retained in U251 KD and U87MG KD tumour cell lines for up to 35 days (*P* ≤ .0001; Figure S7A). The silencing gradually reversed after 35 days in both tumour cell lines.

**FIGURE 5:**
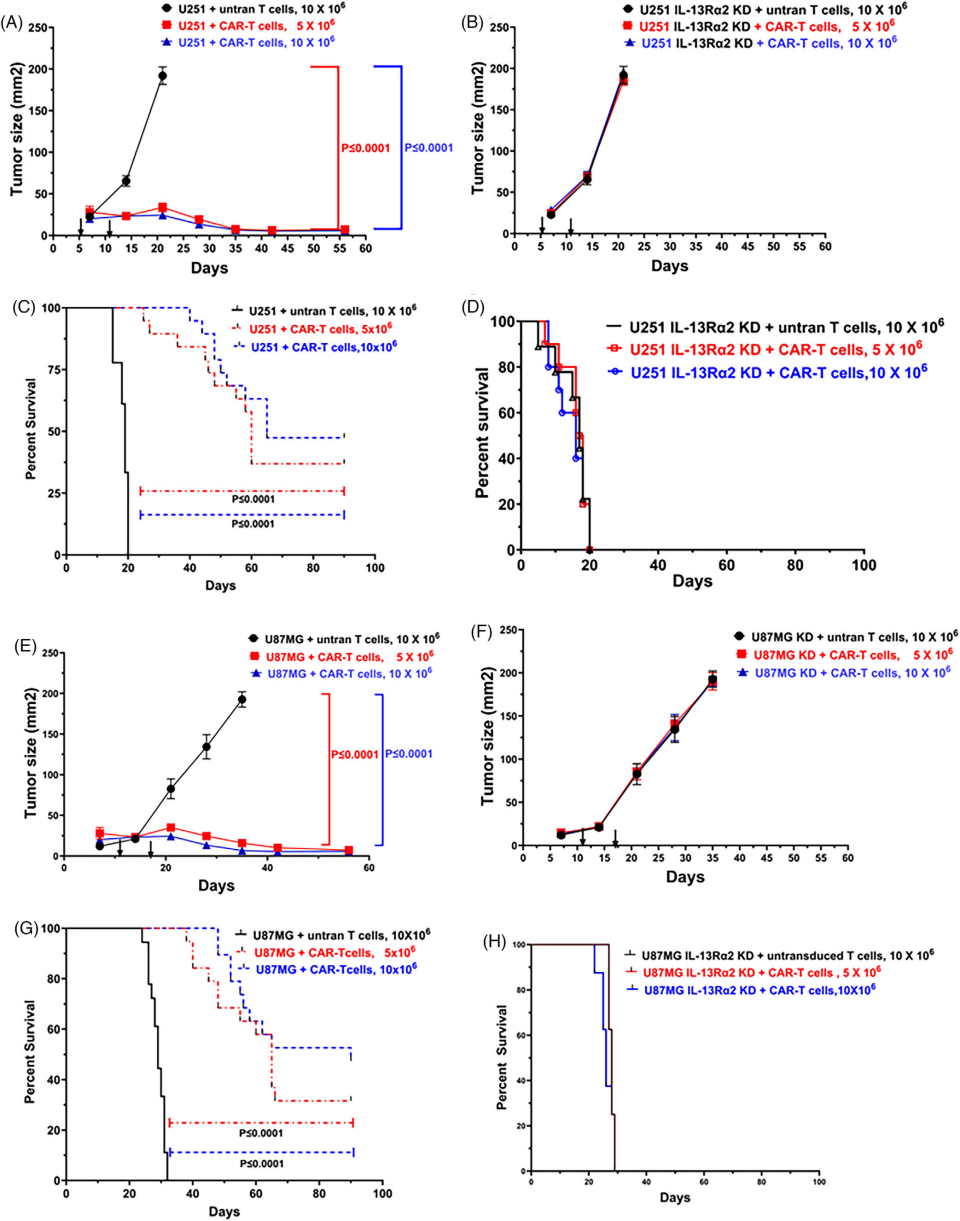
Biological efficacy of CAR‐T cells in vivo xenograft mouse model of human glioblastoma: subcutaneous xenografts of U251 and U87MG GBM tumour s were administered with 5 and 10 × 10^6^ CAR‐T cells by tail vein. Tumour growth was monitored by measuring tumour size at specified time points. CAR‐T cells regressed subcutaneous tumours significantly (*P* ≤ .0001) in a dose‐dependent manner (A,E). CAR‐T cells treated mice survived longer without any general health issues (C,G) compared to mice treated with untransduced T cells. In contrast, mice with U251 IL‐13Rα2 KD and U87MG IL‐13Rα2 xenografts reached 200 mm^2^ similar to U251 or U87MG xenografts (B,F). Similarly, K‐M survival time for U251 and U87 MG IL‐13Rα2 KD xenografts bearing mice with or without CAR‐T treatment was shorter compared to their counterparts (5D,H).

To determine the effectiveness of scFv‐IL‐13Rα2 CAR‐T cells treatment to IL‐13Rα2 positive glioma xenografts in NOG mice, U251, U251‐IL‐13Rα2 knock down, U87MG and U87MG IL‐13Rα2 knock down glioma cells were subcutaneously implanted in NOG female mice. Beginning day 10 of implantation, tumours were palpable, and mice were infused by tail vein with 5 × 10^6^ and 10 × 10^6^ CAR‐T cells having less than 3.6 EU/mL of endotoxin (run #2 and 4 CAR‐T cells were used as shown in Table 3) once a week for 2 weeks. The viral copy number of the CAR+ T cell product administered was 2.5 ± .12/CAR + T cell. Control tumour bearing mice were infused with non‐transduced T cells following the same schedule. As shown in Figure 5A,E, both doses of CAR‐T cells regressed the tumours significantly (*P* ≤ .0001) compared with mice treated with 10 × 10^6^‐untransduced T cells. Interestingly, xenografts grew faster from tumour cells expressing more IL‐13Rα2 receptor sites such as U251 compared to those from lower number of IL‐13Rα2 expressing tumour cells such as U87MG tumour cells. As shown in Figure 5C,G, treated mice with both doses survived longer in U251 and U87 xenograft bearing mice more than 75 days post treatment compared to corresponding untransduced T cell treated mice (*P* ≤ .0001). The control group of mice were euthanised approximately between 4−5 weeks in both U251 and U87MG xenografts for ethical reasons because tumours grew faster reaching to a size of 200 mm^2^. We also observed a similar pattern of tumour growth of U251 KD and U87MG KD xenografts in mice treated with either untransduced T cells or CAR‐T cells as shown in Figure 5B,F. All these mice were euthanised approximately between the same time frame as control group between 18−24 days in both U251KD and U87MG KD xenograft groups (Figure 5D,H) for ethical reasons because tumours reached a maximal allowable tumour size of 200 mm^2^. As shown in Figure S7B, levels of IL‐13Rα2 mRNA were still less than 10% in U251KD and U87MG KD xenografts compared to U251 and U87MG tumours (*P* ≤ .0001) at the end of the experiment. Mice with larger tumours could potentially interfere with mobility and may have difficulty in reaching food or water. Overall health conditions of CAR‐T cells treated mice were better in eating/drinking water habits and mobility activity. All mice remained healthy during the study.

### 3.9 Non‐specific toxicity in mice

General toxicity in non‐tumour bearing mice due to the CAR‐T cell administration was evaluated in non‐tumour bearing NOG mice because mice are not expected to express high levels of IL‐13Rα2 in any organ. Five mice in each group received 5 × 10^6^ and 10 × 10^6^ CAR‐T cells infused by tail vein. The mice were monitored for 90 days. The CAR‐T cell administration showed remarkable safety profile in terms of change in serum enzymes such as aldolase, alanine aminotransferase, CK and serum creatinine (Figure S8B–E) and body weights (Table S1). Two independent experiments showed no significant change in these serum chemistry analytes in the mice nor did they lose body weight. No mortality was observed and both doses of CAR‐T cells were well tolerated (Figure S8A).

## 4 DISCUSSION

In the present study, we describe the discovery, generation and characterisation of a novel scFv‐based CAR‐T cells designed to target IL‐13Rα2 expressing human solid cancer cells. We used codon optimised IL‐13Rα2‐scFv specific phage clones using recombinant human IL‐13Rα2Fc chimeric protein as antigen. This clone is developed from a scFv phagemid library of synthetic v‐gene segments and improved version of V_H_ and V_L_ showing a hyper‐variable amino‐acid structural composition from human synthetic Fab Lox library. Five times higher binding activity of improved scFv # clone 14‐1 could be due to difference of three amino acid residues “HMI” at position 102−104 in CDRH3 and difference of one amino acid “M” at position 225 in CDRL 3. We selected a novel conserved scFv fragment that is involved in optimum antigen binding based on amino acid residue analysis by four different schemes, which revealed a structure based scFv variable regions, defined loop structures that form the CDRs and insertion points inside CDRs exhibiting a hyper‐variable amino acid composition. Using this fragment, we constructed a novel third generation lentiviral CAR‐construct with scFv‐human IL‐13Rα2 as an ectodomain and CD28 cytoplasmic, 4‐1BB and CD3ζ as endodomains.

In the present study, we demonstrate that engineered T cells expressing scFv‐huIL‐13Rα2 CAR proliferate, bind target antigen, and exhibit the cytotoxic activity to IL‐13Rα2 positive tumour cells, but not to IL‐13Rα2 negative tumour cells or IL‐13Rα2 gene silenced tumour cells. IL‐13Rα2‐CARs also induced IFN‐γ secretion in a strictly IL‐13Rα2‐dependent manner. The dose regimen of the CAR‐T cell therapy in the pre‐clinical mouse model of solid human cancer is highly debatable because of tumour heterogeneity and complex tumour microenvironment (TME), which vary from tumour type, stage and site. It is also not clearly understood if single or multiple infusion of CAR‐T cells are required for an effective or achievable therapeutic response in pre‐clinical or clinical settings. We used two different doses of CAR‐T cell drug product in our initial phase of study to test the proof‐of‐principle. Based on our data, the CAR‐T drug product, which had permissible endotoxin levels and vector copy number per CAR+ T cell, was found effective in regressing the U251 and U87MG xenografts of human glioma. Our future study aims at studying the in vivo effectiveness of the CAR‐T drug product administered via different routes of administration. Together, these results indicate that scFv‐IL‐13Rα2 CAR‐T cells can be highly effective against IL‐13Rα2+ human solid malignancies. Our next step of the research is to generate a murine counter part of CAR‐T cells with murine scFv‐IL‐13Rα2 as a transgene and test its quality attributes in vitro and in vivo in syngeneic mouse models using murine IL‐13Rα2 positive glioma cell lines such as GL121 and CTA2. We also plan to examine the relative effectiveness of this murine CAR‐T cell product with its human counterpart in the subcutaneous as well as orthotopic tumour models after administration through different routes, which would then justify the intracranial model in subsequent phases of the research. Further, this study will include the development of clinical grade scFv IL‐13Rα2‐lentiviral vector and manufacturing CAR‐T drug product in cGMP conditions that will have mutated WPRE element and permissible vector copy number.

Other studies have reported targeting of IL‐13Rα2 positive cancer by CAR‐T cells. Pituch et al. generated a CAR‐T construct by using scFv derived from an IL‐13Rα2 specific monoclonal antibody fused with a murine IgG leader peptide, the hinge region of murine IgG1, the CD28 transmembrane domain, and the CD28 and CD3ζ signalling domains to study the therapeutic potential of CAR‐T cells in a murine model of glioma.^42^ In a different study of scFv‐based CARs, Krenciute et al. developed CARs using the same scFv antigen binding domain with short or long spacer regions and CD28TM, CD28 endodomain, either 4‐1BB or OX40 and CD3ξ endodomains.^43^ In addition, both studies used the murine scFv component in their CAR constructs. However, we cloned a high binding activity scFv fragment from a human synthetic library. We expect that there will be minimal or no immune response against the scFv fragment in CAR‐T cell product. Most frequently, portions or entire mouse mAb have been used to make immunotherapeutic agents to treat cancer in pre‐clinical investigational studies.^44^, ^45^ Because human anti‐mouse antibodies form complexes with circulating therapeutic mono‐clonal antibody or fragment/s, it has been quite difficult to achieve efficacy endpoint of circulating therapeutic monoclonal antibodies or immunotoxin.^46^, ^47^ We engineered several changes in our CAR‐T construct to minimise such complexity or restrictions or adverse side effects. These chimeric antibodies or ‘‘humanised’’ antibodies have been introduced to decrease or minimise the hypersensitive reactions to xenogeneic proteins in the host.^48^, ^49^ Additionally, we used costimulatory 4‐1BB sequences in the intracellular domain of CAR‐T cells for additional signalling for its ability to enhance T‐cell persistence and tumour localisation.^50^, ^51^, ^52^ Thus, it is possible that our novel CAR‐T cells could persist longer and mediate prolonged anti‐tumour activity in the localised TME due to the lack of murine components and our future studies will address this possibility.

In another study, IL‐13Rα2 specific CAR‐T cells have been generated, which do not have scFv, but a membrane‐tethered IL13‐mutein with one or two amino acid substitutions to preferentially redirect T cells to IL‐l3Rα2.^53^, ^54^, ^55^ It was proposed that CAR‐T cells expressing IL‐13‐mutein do not recognise IL‐13Rαl ‐on target cells.^56^ We have shown that IL‐13 can efficiently cause activation of STAT 6 protein in transfected with the IL‐13Rα1 and IL‐4Rα chains.^57^ Additional studies have shown that IL13‐mutein do recognise IL‐13Rα1‐positive targets, which could lead to off‐target toxicities.^13^, ^15^, ^55^, ^58^ Because of ubiquitous nature of expression of IL‐13Rα1, CARs with membrane tethered IL13‐mutein would exhibit off target toxicity in normal tissues.^55^ We believe that the scFv‐based CAR‐T cell therapy may have advantage in developing IL‐13Rα2 targeted CAR‐T cell therapy product. Our ongoing studies will address the possibility that our newly engineered anti‐IL‐13 Rα2 construct will improve tumour specificity and reduce potential for off target effects due to expression of IL‐13 Rα1 on normal tissues.

Interestingly, IL‐13Rα2 is overexpressed on numerous human solid cancers, but its expression is not homogenous. In general, a pocket of cells is strongly positive, while others are negative for IL‐13Rα2 expression. Thus, it is highly desirable to uniformly upregulate IL‐13Rα2 expression in tumours for optimal targeting by CAR‐T cells. We have previously reported that histone deacetylase (HDAC) inhibitors dramatically enhanced IL‐13Rα2 expression in receptor‐negative pancreatic cancer cells and in addition, HDAC inhibitors enhanced expression of IL‐13Rα2 in IL‐13Rα2 positive tumours.^59^ In contrast, HDAC inhibition did not increase IL‐13Rα2 in normal cell lines or tissues. Furthermore, HDAC inhibitors dramatically sensitised pancreatic cancer cells to IL‐13‐PE cytotoxicity in vitro and in animal models of human pancreatic cancer.^59^ Similarly, we have also demonstrated that calcitonin gene related peptides (CGRPs) induce and upregulate expression of IL‐13Rα2 in prostate cancers in vitro and in vivo.^40^ Thus, we hypothesise that pre‐treatment of hosts either with HDAC‐inhibitors or CGRPs will synergise with CAR‐T cells in targeting IL‐13Rα2 positive solid cancer for a better anti‐ tumour effect. In this regard, combination therapies consisting of concomitant use of the CAR‐T cell products with steroids or alternative agents such as bevacizumab, corticorelin and Boswellic acids are newly emerged areas of research where much effort is needed.^60^ Studies that balance and optimise the aggressive anti‐tumour effects of CAR‐T cells with the opposing physiological effects of immunosuppression and oedema suppression, reparative effects of other alternative agents and additional anti‐tumour effects of agents such as bevacizumab or anti‐tumour oncolytic agents^61^ may be beneficial for patients seeking cures as opposed to palliative measures.

One of the major impediments in targeting CAR‐T cells to solid tumour s is the presence of tumour stroma and complex TME. Typically, TMEs are comprised of tumour cells and together with a variety of other cell types such as tumour‐associated macrophages, fibroblasts, endothelial cells, regulatory T cells, myeloid derived suppressor cells (MDSC) and immune B cells.^18^ MDSCs are considered to be the most significant suppressor cells in the cancer‐bearing host.^62^, ^63^ Since we have demonstrated that MDSCs and regulatory T cells express IL‐13Rα2,^64^ it is possible that CAR‐T cells with a high binding targeting structure (scFv‐IL‐13Rα2 as a payload) will have a better chance of demonstrating a robust anti‐tumour effect. While investigating this possibility, it will be prudent to also consider that there could be an increase in toxicity due to killing of on‐target, off‐tumour cell types and increased CRS/neurotoxicity at these sites.

In summary, we have demonstrated in the present study that scFv‐IL‐13Rα2‐CAR‐T cells can efficiently target IL‐13Rα2 overexpressing human tumour cells in vitro and in pre‐clinical models of human glioma cancers as the binding of our newer clone #14‐1 in scFv‐IL‐13Rα2‐ CAR construct is ∼5 times more than the previous clone #4.^30^, ^65^ The fast initial anti‐tumour responses in vivo may be mostly because of the interaction of CAR‐T cells with the IL‐13Rα2 positive tumours‐targeted component of the response. Although we do not have direct comparative in vivo data in the xenograft model, our previous data from clone 4^30^, ^65^ show that tumour persists longer and grows faster than what we observed in the current experiments, which is a part of our future research.

We reveal IL‐13Rα2 down‐modulation on tumour cells and induction of tumour cell killing as an important mechanism of tumour regression after CAR‐T cell contact mediated by interaction of CAR‐T cells with IL‐13Rα2 and its internalisation. Importantly, TME, route of administration and its bio‐distribution are important key factors that may determine overall anti‐ tumour effects of CAR‐T cells in vivo in pre‐clinical and clinical settings. Our present study provides an insight on designing an effective and novel anti‐cancer agent in form of the CAR‐T cell therapy and may prove useful after studying its therapeutic properties in further pre‐clinical and clinical studies. Also, further study of the mechanism of action and appropriate route of administration in a variety of pre‐clinical models of human cancers are critical and important as scFV‐IL‐13Rα2‐CAR‐T cells specifically bind to receptor positive cell and kill them, which may help developing more effective therapeutic strategies in the future.

## AUTHOR CONTRIBUTIONS

Pamela Leland, Heba Degheidy and Ashley Lea performed experiments acquired analysed data. Steven R. Bauer, Raj K. Puri and Bharat H. Joshi designed the study and wrote the manuscript. Raj K. Puri and Bharat H. Joshi conceived the project and supervised the study. All authors reviewed and edited the manuscript.

## CONFLICT OF INTEREST STATEMENT

The authors declare no conflicts of interest. The following patent application is related to the work presented in this paper: PCT Patent Application No. PCT/US2022/023112, Inventors: RKP; BHJ.

## FUNDING INFORMATION

None.

## ETHICS APPROVAL

All animal studies were reviewed and approved by the Center for Biologics Evaluation and Research, FDA, Animal Care and Use Committee (IUCAC Protocol 2003‐08).

## CONSENT FOR PUBLICATION

All authors approved the manuscript.

## Supporting information


**Figure S1** (A) Schematic diagram of generating scFv‐IL‐13Rα2 antibody fragment by phage display technique showing step‐by‐step production of scFv‐IL‐13Rα2.

(B) Comparative assessment of the binding activity of previous clone scFv‐IL‐13Rα2 (# 4 control) with the present clone # 14‐1 derived after biopanning # 14‐1 by ELISA assay as described in material and methods.

(C) Amino acid sequence alignment of clones # 4 (previous clone) vs. newly improved clone # 14‐1 describing differences in amino acid residues of CDRH3 (highlighted in red) and CDRL3 (highlighted in blue).

**Figure S2** (A) DNA sequence of scFv‐IL‐13Rα2 construct and its domains.

(B). Amino acid sequence and its different domains.

**Figure S3** (A) Amino acid numbering of heavy chain residues by Kabat numbering scheme.

(B) Analysis of heavy chain amino acids, their numbers and length in each region.

(C) Loop structure of CDRs and amino acid residue position number—heavy chain: shown in blue circle are hydrophobic residues, anchor position, conserved amino acids AA, β‐strand direction.

**Figure S4** (A) Amino acid numbering of Light chain residues with Kabat numbering scheme.

(B) Analysis of light chain amino acids, their numbers and length in each region.

(C) Loop structure of CDRs and amino acid residue position number—light chain: shown in blue circle are hydrophobic residues, anchor position, conserved amino acids AA, β‐strand direction.

**Figure S5** (A) Distribution of heavy chain amino acids and their respective frequency.

(B) Distribution of light chain amino acids and their respective frequency.

**Figure S6** (A) Numbering of scFv residue sequence for a comparative alignment analysis—heavy chain. Alignment of heavy chain residues according to IMGT, Kabat, Chothia and Martin numbering schemes. In red CDR1, orange CDR2 and purple CDR3 are shown.

(B) Numbering of scFv residue sequence for a comparative alignment analysis— light chain. Alignment of heavy chain residues according to IMGT, Kabat, Chothia and Martin numbering schemes. In blue CDR1, light green CDR2 and green CDR3 are shown.

**Figure S7** (A) Analysis of RT‐qPCR analysis for IL‐13Rα2 RNA expression from U251 and U87MG glioma cell lines at different time points up to 49 days. Each value is a mean ± SD (standard deviation) of four independent experiments performed in triplicate. (B) IL‐13Rα2 gene silencing was examined by RT‐qPCR in U251 and U87MG xenograft tumour tissues at the end of the experiment. Each value is a mean ± SD of four independent experiments performed in triplicate.

**Figure S8** (A) K‐M survival of non‐tumour bearing mice treated with untransduced and CAR‐T cells. Each datum is an average ± SD of two independent experiments each consisted of six mice: (B) serum chemistry analysis for the serum aldolase activity, (C) serum ALT activity, (D) serum CK activity, and (E) serum creatinine from mice sera collected on day 0 and 90. Each value represents mean ± SD of triplicate analysis of the samples from two independent experiments.

Supporting Information

## Data Availability

The data supporting the findings of this study are available on request from the corresponding author.

## References

[1] Neelapu SS , Locke FL , Bartlett NL , et al. Axicabtagene ciloleucel CAR T‐cell therapy in refractory large B‐cell lymphoma. N Engl J Med. 2017;377:2531‐2544. 29226797 10.1056/NEJMoa1707447 PMC5882485

[2] Davenport AJ , Cross RS , Watson KA , et al. Chimeric antigen receptor T cells form nonclassical and potent immune synapses driving rapid cytotoxicity. Proc Natl Acad Sci U S A. 2018;115:E2068‐E2076. 29440406 10.1073/pnas.1716266115 PMC5834689

[3] Schuster SJ , Svoboda J , Chong EA , et al. Chimeric antigen receptor T cells in refractory B‐cell lymphomas. N Engl J Med. 2017;377:2545‐2554. 29226764 10.1056/NEJMoa1708566 PMC5788566

[4] FDA: FDA approval brings first gene therapy to the United States. FDA; 2017.

[5] FDA: FDA approves First Cell‐Based Gene Therapy For Adult Patientswith Relapsed or Refractory MCL. FDA; 2020.

[6] Lim WA , June CH . The principles of engineering immune cells to treat cancer. Cell. 2017;168:724‐740. 28187291 10.1016/j.cell.2017.01.016 PMC5553442

[7] Ramos CA , Rouce R , Robertson CS , et al. In vivo fate and activity of second‐ versus third‐generation CD19‐specific CAR‐T cells in B cell non‐Hodgkin's lymphomas. Mol Ther. 2018;26:2727‐2737. 30309819 10.1016/j.ymthe.2018.09.009 PMC6277484

[8] Ramos CA , Heslop HE , Brenner MK . CAR‐T cell therapy for lymphoma. Annu Rev Med. 2016;67:165‐183. 26332003 10.1146/annurev-med-051914-021702 PMC4732525

[9] Sadelain M . CD19 CAR T cells. Cell. 2017;171:1471. 29245005 10.1016/j.cell.2017.12.002

[10] Curran KJ , Margossian SP , Kernan NA , et al. Toxicity and response after CD19‐specific CAR T‐cell therapy in pediatric/young adult relapsed/refractory B‐ALL. Blood. 2019;134:2361‐2368. 31650176 10.1182/blood.2019001641 PMC6933289

[11] Davila ML , Sadelain M . Biology and clinical application of CAR T cells for B cell malignancies. Int J Hematol. 2016;104:6‐17. 27262700 10.1007/s12185-016-2039-6 PMC5512169

[12] Joshi BH , Hogaboam C , Dover P , Husain SR , Puri RK . Role of interleukin‐13 in cancer, pulmonary fibrosis, and other T(H)2‐type diseases. Vitam Horm. 2006;74:479‐504. 17027527 10.1016/S0083-6729(06)74019-5

[13] Suzuki A , Leland P , Joshi BH , Puri RK . Targeting of IL‐4 and IL‐13 receptors for cancer therapy. Cytokine. 2015;75:79‐88. 26088753 10.1016/j.cyto.2015.05.026

[14] Joshi BH , Plautz GE , Puri RK . Interleukin‐13 receptor alpha chain: a novel tumor‐associated transmembrane protein in primary explants of human malignant gliomas. Cancer Res. 2000;60:1168‐1172. 10728667

[15] Knudson KM , Hwang S , McCann MS , Joshi BH , Husain SR , Puri RK . Recent advances in IL‐13Ralpha2‐directed cancer immunotherapy. Front Immunol. 2022;13:878365. 35464460 10.3389/fimmu.2022.878365 PMC9023787

[16] Wykosky J , Gibo DM , Stanton C , Debinski W . Interleukin‐13 receptor alpha 2, EphA2, and Fos‐related antigen 1 as molecular denominators of high‐grade astrocytomas and specific targets for combinatorial therapy. Clin Cancer Res. 2008;14:199‐208. 18172271 10.1158/1078-0432.CCR-07-1990

[17] Zeng J , Zhang J , Yang YZ , et al. IL13RA2 is overexpressed in malignant gliomas and related to clinical outcome of patients. Am J Transl Res. 2020;12:4702‐4714. 32913543 PMC7476143

[18] Beard RE , Abate‐Daga D , Rosati SF , et al. Gene expression profiling using nanostring digital RNA counting to identify potential target antigens for melanoma immunotherapy. Clin Cancer Res. 2013;19:4941‐4950. 24021875 10.1158/1078-0432.CCR-13-1253 PMC3778100

[19] Chiaramonte MG , Mentink‐Kane M , Jacobson BA , et al. Regulation and function of the interleukin 13 receptor alpha 2 during a T helper cell type 2‐dominant immune response. J Exp Med. 2003;197:687‐701. 12642601 10.1084/jem.20020903 PMC2193852

[20] Donaldson DD , Whitters MJ , Fitz LJ , et al. The murine IL‐13 receptor alpha 2: molecular cloning, characterization, and comparison with murine IL‐13 receptor alpha 1. J Immunol. 1998;161:2317‐2324. 9725226

[21] Mentink‐Kane MM , Wynn TA . Opposing roles for IL‐13 and IL‐13 receptor alpha 2 in health and disease. Immunol Rev. 2004;202:191‐202. 15546394 10.1111/j.0105-2896.2004.00210.x

[22] Jaen M , Martin‐Regalado A , Bartolome RA , Robles J , Casal JI . Interleukin 13 receptor alpha 2 (IL13Ralpha2): expression, signaling pathways and therapeutic applications in cancer. Biochim Biophys Acta Rev Cancer. 2022;1877:188802. 36152905 10.1016/j.bbcan.2022.188802

[23] Gardner R , Wu D , Cherian S , et al. Acquisition of a CD19‐negative myeloid phenotype allows immune escape of MLL‐rearranged B‐ALL from CD19 CAR‐T‐cell therapy. Blood. 2016;127:2406‐2410. 26907630 10.1182/blood-2015-08-665547 PMC4874221

[24] Jacoby E , Nguyen SM , Fountaine TJ , et al. CD19 CAR immune pressure induces B‐precursor acute lymphoblastic leukaemia lineage switch exposing inherent leukaemic plasticity. Nat Commun. 2016;7:12320. 27460500 10.1038/ncomms12320 PMC4974466

[25] Yeku OO , Purdon TJ , Koneru M , Spriggs D , Brentjens RJ . Armored CAR T cells enhance antitumor efficacy and overcome the tumor microenvironment. Sci Rep. 2017;7:10541. 28874817 10.1038/s41598-017-10940-8 PMC5585170

[26] Fichtner‐Feigl S , Strober W , Kawakami K , Puri RK , Kitani A . IL‐13 signaling through the IL‐13alpha2 receptor is involved in induction of TGF‐beta1 production and fibrosis. Nat Med. 2006;12:99‐106. 16327802 10.1038/nm1332

[27] Han J , Puri RK . Analysis of the cancer genome atlas (TCGA) database identifies an inverse relationship between interleukin‐13 receptor alpha1 and alpha2 gene expression and poor prognosis and drug resistance in subjects with glioblastoma multiforme. J Neurooncol. 2018;136:463‐474. 29168083 10.1007/s11060-017-2680-9 PMC5805806

[28] Joshi BH , Kawakami K , Leland P , Puri RK . Heterogeneity in interleukin‐13 receptor expression and subunit structure in squamous cell carcinoma of head and neck: differential sensitivity to chimeric fusion proteins comprised of interleukin‐13 and a mutated form of Pseudomonas exotoxin. Clin Cancer Res. 2002;8:1948‐1956. 12060640

[29] Joshi BH , Puri RK . Optimization of expression and purification of two biologically active chimeric fusion proteins that consist of human interleukin‐13 and Pseudomonas exotoxin in *Escherichia coli* . Protein Expr Purif. 2005;39:189‐198. 15642470 10.1016/j.pep.2004.10.012

[30] Kioi M , Seetharam S , Puri RK . Targeting IL‐13Ralpha2‐positive cancer with a novel recombinant immunotoxin composed of a single‐chain antibody and mutated Pseudomonas exotoxin. Mol Cancer Ther. 2008;7:1579‐1587. 18566228 10.1158/1535-7163.MCT-07-2131

[31] Mitra A , Barua A , Huang L , Ganguly S , Feng Q , He B . From bench to bedside: the history and progress of CAR T cell therapy. Front Immunol. 2023;14:1188049. 37256141 10.3389/fimmu.2023.1188049 PMC10225594

[32] Roselli E , Boucher JC , Li G , et al. 4‐1BB and optimized CD28 co‐stimulation enhances function of human mono‐specific and bi‐specific third‐generation CAR T cells. J Immunother Cancer. 2021;9:e003354. 10.1136/jitc-2021-003354 PMC8552146 34706886

[33] Sterner RC , Sterner RM . CAR‐T cell therapy: current limitations and potential strategies. Blood Cancer J. 2021;11:69. 33824268 10.1038/s41408-021-00459-7 PMC8024391

[34] DeRenzo C , Krenciute G , Gottschalk S . The landscape of CAR T cells beyond acute lymphoblastic leukemia for pediatric solid tumors. Am Soc Clin Oncol Educ Book. 2018;38:830‐837. 30231350 10.1200/EDBK_200773 PMC11613507

[35] Bradbury AR , Marks JD . Antibodies from phage antibody libraries. J Immunol Methods. 2004;290:29‐49. 15261570 10.1016/j.jim.2004.04.007

[36] Marks JD , Hoogenboom HR , Bonnert TP , McCafferty J , Griffiths AD , Winter G . By‐passing immunization. Human antibodies from V‐gene libraries displayed on phage. J Mol Biol. 1991;222:581‐597. 1748994 10.1016/0022-2836(91)90498-u

[37] Marks JD , Tristem M , Karpas A , Winter G . Oligonucleotide primers for polymerase chain reaction amplification of human immunoglobulin variable genes and design of family‐specific oligonucleotide probes. Eur J Immunol. 1991;21:985‐991. 2019291 10.1002/eji.1830210419

[38] Dunbar J , Deane CM . ANARCI: antigen receptor numbering and receptor classification. Bioinformatics. 2016;32:298‐300. 26424857 10.1093/bioinformatics/btv552 PMC4708101

[39] Leland P , Kumar D , Nimmagadda S , Bauer SR , Puri RK , Joshi BH . Characterization of chimeric antigen receptor modified T cells expressing scFv‐IL‐13Ralpha2 after radiolabeling with (89)Zirconium oxine for PET imaging. J Transl Med. 2023;21:367. 37286997 10.1186/s12967-023-04142-2 PMC10246418

[40] Joshi BH , Leland P , Calvo A , Green JE , Puri RK . Human adrenomedullin up‐regulates interleukin‐13 receptor alpha2 chain in prostate cancer in vitro and in vivo: a novel approach to sensitize prostate cancer to anticancer therapy. Cancer Res. 2008;68:9311‐9317. 19010904 10.1158/0008-5472.CAN-08-2810 PMC6944210

[41] Barczak W , Suchorska W , Rubis B , Kulcenty K . Universal real‐time PCR‐based assay for lentiviral titration. Mol Biotechnol. 2015;57:195‐200. 25370825 10.1007/s12033-014-9815-4 PMC4298670

[42] Pituch KC , Miska J , Krenciute G , et al. Adoptive transfer of IL13Ralpha2‐specific chimeric antigen receptor T cells creates a pro‐inflammatory environment in glioblastoma. Mol Ther. 2018;26:986‐995. 29503195 10.1016/j.ymthe.2018.02.001 PMC6079480

[43] Krenciute G , Krebs S , Torres D , et al. Characterization and functional analysis of scFv‐based chimeric antigen receptors to redirect T cells to IL13Ralpha2‐positive glioma. Mol Ther. 2016;24:354‐363. 26514825 10.1038/mt.2015.199 PMC4817815

[44] Kreitman RJ . Immunotoxins for targeted cancer therapy. AAPS J. 2006;8:E532‐551. 17025272 10.1208/aapsj080363 PMC2761061

[45] Pastan I , Hassan R , FitzGerald DJ , Kreitman RJ . Immunotoxin treatment of cancer. Annu Rev Med. 2007;58:221‐237. 17059365 10.1146/annurev.med.58.070605.115320

[46] Badger CC , Anasetti C , Davis J , Bernstein ID . Treatment of malignancy with unmodified antibody. Pathol Immunopathol Res. 1987;6:419‐434. 3333188 10.1159/000157067

[47] Khazaeli MB , Conry RM , LoBuglio AF . Human immune response to monoclonal antibodies. J Immunother Emphasis Tumor Immunol. 1994;15:42‐52. 8110730 10.1097/00002371-199401000-00006

[48] Mountain A , Adair JR . Engineering antibodies for therapy. Biotechnol Genet Eng Rev. 1992;10:1‐142. 1301737 10.1080/02648725.1992.10647886

[49] Wright A , Shin SU , Morrison SL . Genetically engineered antibodies: progress and prospects. Crit Rev Immunol. 1992;12:125‐168. 1476621

[50] Kudo K , Imai C , Lorenzini P , et al. T lymphocytes expressing a CD16 signaling receptor exert antibody‐dependent cancer cell killing. Cancer Res. 2014;74:93‐103. 24197131 10.1158/0008-5472.CAN-13-1365

[51] Song DG , Ye Q , Carpenito C , et al. In vivo persistence, tumor localization, and antitumor activity of CAR‐engineered T cells is enhanced by costimulatory signaling through CD137 (4‐1BB). Cancer Res. 2011;71:4617‐4627. 21546571 10.1158/0008-5472.CAN-11-0422 PMC4140173

[52] Kadayakkara DK , Damodaran K , Hitchens TK , Bulte JW , Ahrens ET . (19)F spin‐lattice relaxation of perfluoropolyethers: dependence on temperature and magnetic field strength (7.0‐14.1T). J Magn Reson. 2014;242:18‐22. 24594752 10.1016/j.jmr.2014.01.014 PMC4008704

[53] Brown CE , Badie B , Barish ME , et al. Bioactivity and safety of IL13Ralpha2‐redirected chimeric antigen receptor CD8+ T cells in patients with recurrent glioblastoma. Clin Cancer Res. 2015;21:4062‐4072. 26059190 10.1158/1078-0432.CCR-15-0428 PMC4632968

[54] Brown CE , Starr R , Aguilar B , et al. Stem‐like tumor‐initiating cells isolated from IL13Ralpha2 expressing gliomas are targeted and killed by IL13‐zetakine‐redirected T Cells. Clin Cancer Res. 2012;18:2199‐2209. 22407828 10.1158/1078-0432.CCR-11-1669 PMC3578382

[55] Krebs S , Chow KK , Yi Z , et al. T cells redirected to interleukin‐13Ralpha2 with interleukin‐13 mutein–chimeric antigen receptors have anti‐glioma activity but also recognize interleukin‐13Ralpha1. Cytotherapy. 2014;16:1121‐1131. 24841514 10.1016/j.jcyt.2014.02.012 PMC4087074

[56] Kahlon KS , Brown C , Cooper LJ , Raubitschek A , Forman SJ , Jensen MC . Specific recognition and killing of glioblastoma multiforme by interleukin 13‐zetakine redirected cytolytic T cells. Cancer Res. 2004;64:9160‐9166. 15604287 10.1158/0008-5472.CAN-04-0454

[57] Kawakami K , Taguchi J , Murata T , Puri RK . The interleukin‐13 receptor alpha2 chain: an essential component for binding and internalization but not for interleukin‐13‐induced signal transduction through the STAT6 pathway. Blood. 2001;97:2673‐2679. 11313257 10.1182/blood.v97.9.2673

[58] Kong S , Sengupta S , Tyler B , et al. Suppression of human glioma xenografts with second‐generation IL13R‐specific chimeric antigen receptor‐modified T cells. Clin Cancer Res. 2012;18:5949‐5960. 22966020 10.1158/1078-0432.CCR-12-0319 PMC4337849

[59] Fujisawa T , Joshi BH , Puri RK . Histone modification enhances the effectiveness of IL‐13 receptor targeted immunotoxin in murine models of human pancreatic cancer. J Transl Med. 2011;9:37. 21477288 10.1186/1479-5876-9-37 PMC3096924

[60] Goldman M , Lucke‐Wold B , Martinez‐Sosa M , et al. Steroid utility, immunotherapy, and brain tumor management: an update on conflicting therapies. Explor Target Antitumor Ther. 2022;3:659‐675. 36338521 10.37349/etat.2022.00106 PMC9630032

[61] Reddy R , Yan SC , Hasanpour Segherlou Z , et al. Oncolytic viral therapy: a review and promising future directions. J Neurosurg. 2024;140:319‐327. 37877961 10.3171/2023.6.JNS23243

[62] Ostrand‐Rosenberg S . Tolerance and immune suppression in the tumor microenvironment. Cell Immunol. 2016;299:23‐29. 26435343 10.1016/j.cellimm.2015.09.011 PMC4698223

[63] Umansky V , Sevko A . Tumor microenvironment and myeloid‐derived suppressor cells. Cancer Microenviron. 2013;6:169‐177. 23242672 10.1007/s12307-012-0126-7 PMC3717060

[64] Hall B , Nakashima H , Sun ZJ , et al. Targeting of interleukin‐13 receptor alpha2 for treatment of head and neck squamous cell carcinoma induced by conditional deletion of TGF‐beta and PTEN signaling. J Transl Med. 2013;11:45. 23421960 10.1186/1479-5876-11-45 PMC3598213

[65] Kioi M , Seetharam S , Puri RK . N‐linked glycosylation of IL‐13R alpha2 is essential for optimal IL‐13 inhibitory activity. FASEB J. 2006;20:2378‐2380. 17023392 10.1096/fj.06-5995fje

