# Supplementary figures and images for: Identification and characterisation of novel CAR‐T cells to target IL13Rα2 positive human glioma in vitro and in vivo
Source: Clin Transl Med. 2024 Apr 29;14(5):e1664. doi: 10.1002/ctm2.1664 (PMC11058282; doi:10.1002/ctm2.1664)

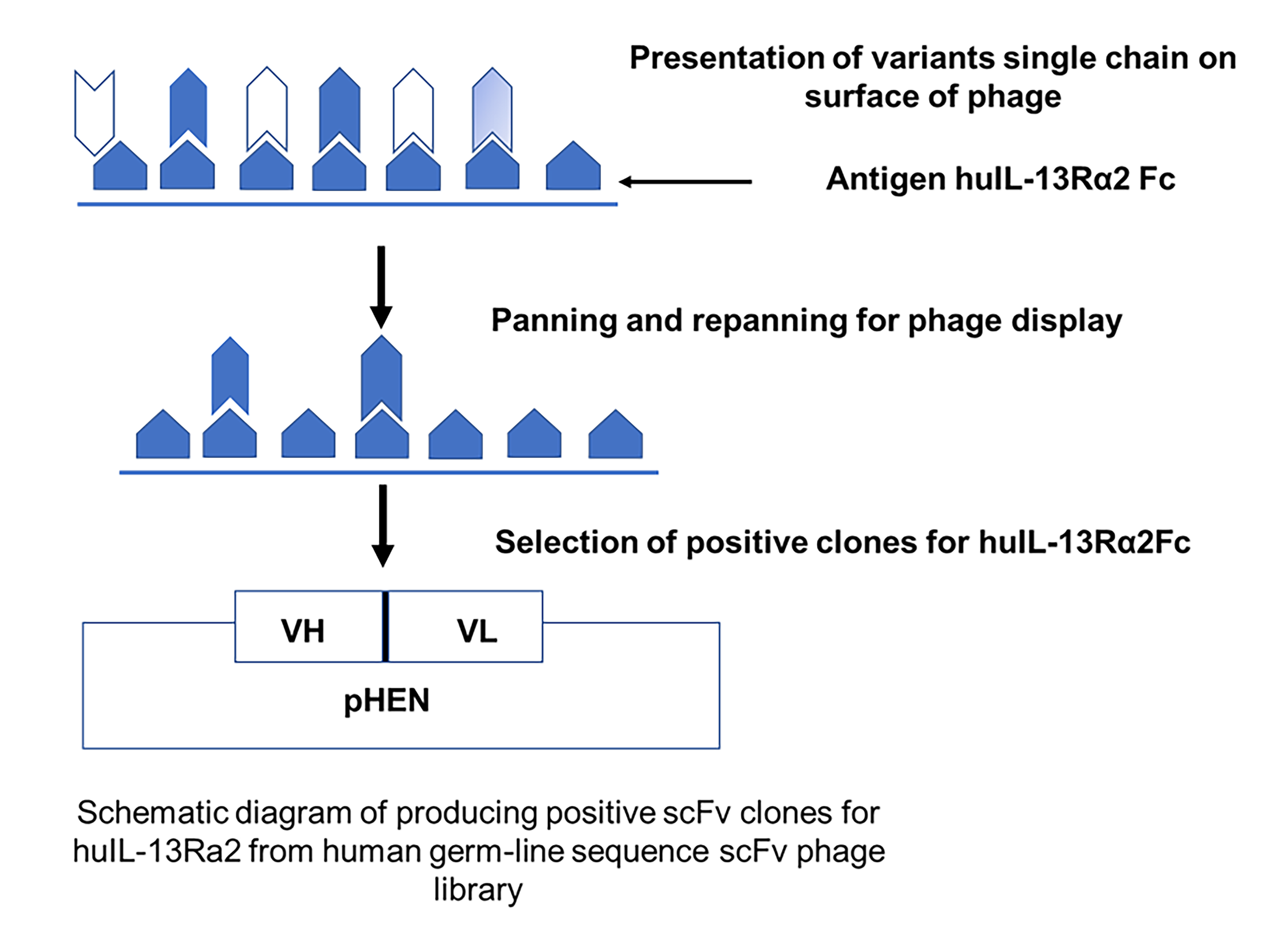

Supplement: Supplementary file 1 — Figure S1 (A) Schematic diagram of generating scFv‐IL‐13Rα2 antibody fragment by phage display technique showing step‐by‐step production of scFv‐IL‐13Rα2. [file CTM2-14-e1664-s016.TIF]

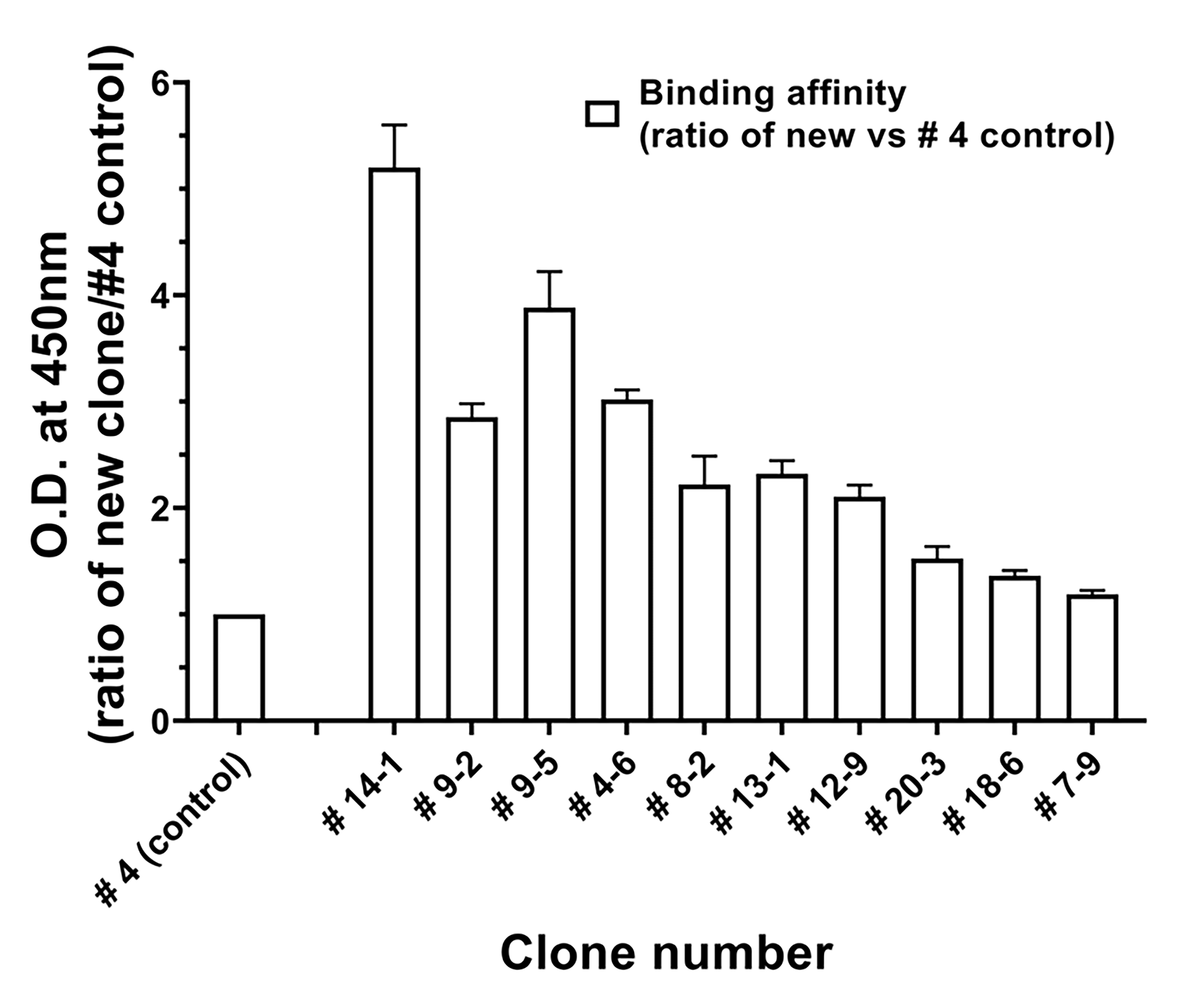

Supplement: Supplementary file 2 — (B) Comparative assessment of the binding activity of previous clone scFv‐IL‐13Rα2 (# 4 control) with the present clone # 14‐1 derived after biopanning # 14‐1 by ELISA assay as described in material and methods. [file CTM2-14-e1664-s010.TIF]

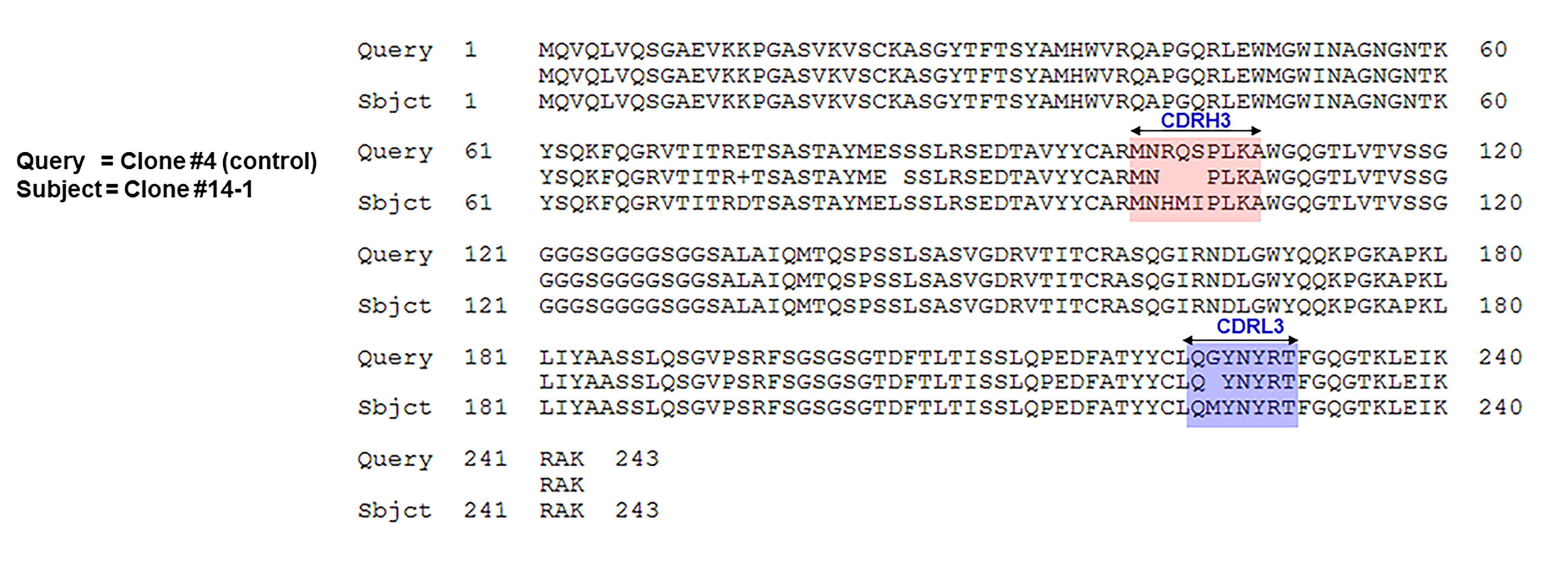

Supplement: Supplementary file 3 — (C) Amino acid sequence alignment of clones # 4 (previous clone) vs. newly improved clone # 14‐1 describing differences in amino acid residues of CDRH3 (highlighted in red) and CDRL3 (highlighted in blue). [file CTM2-14-e1664-s018.TIF]

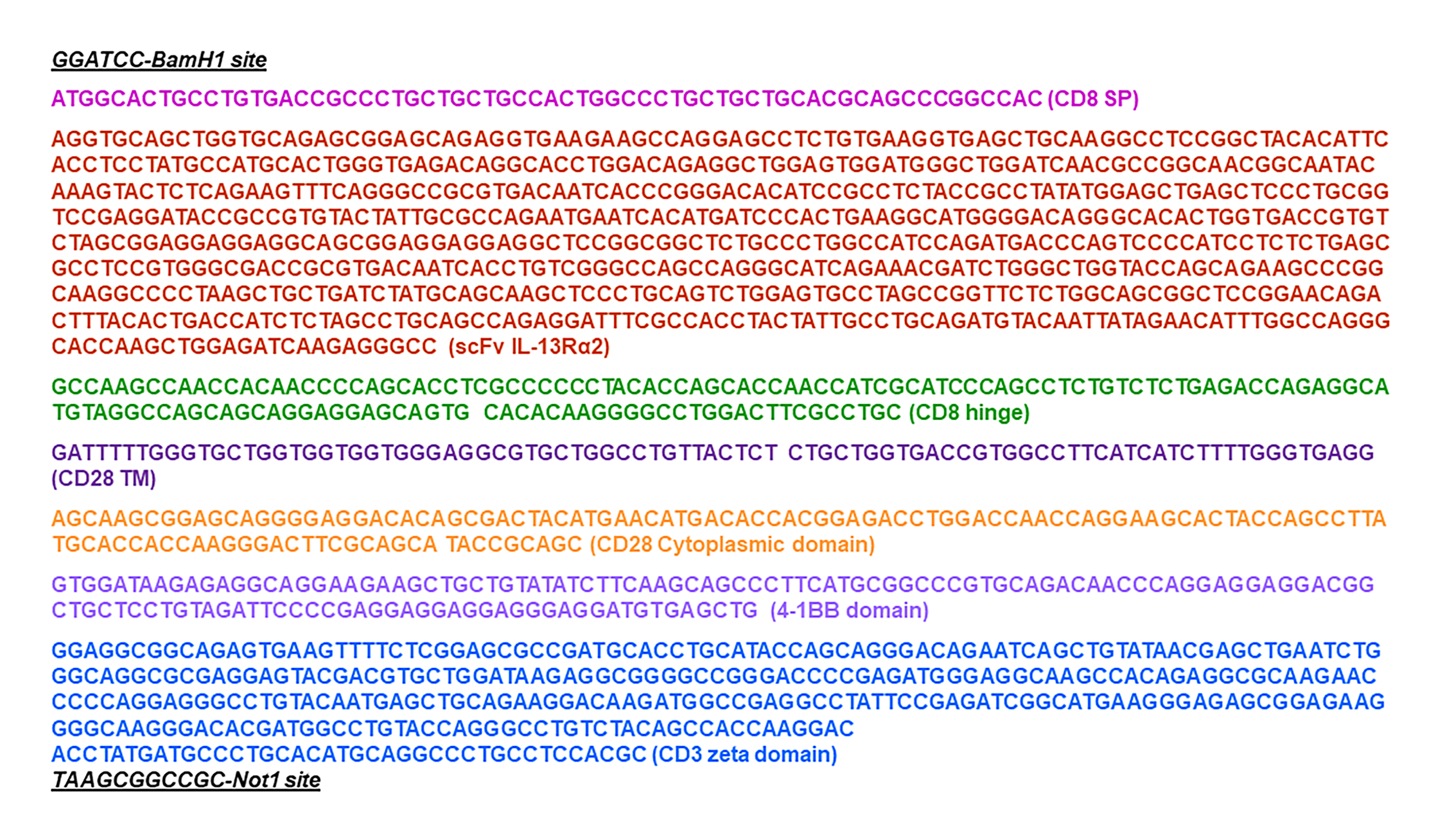

Supplement: Supplementary file 4 — Figure S2 (A) DNA sequence of scFv‐IL‐13Rα2 construct and its domains. [file CTM2-14-e1664-s014.TIF]

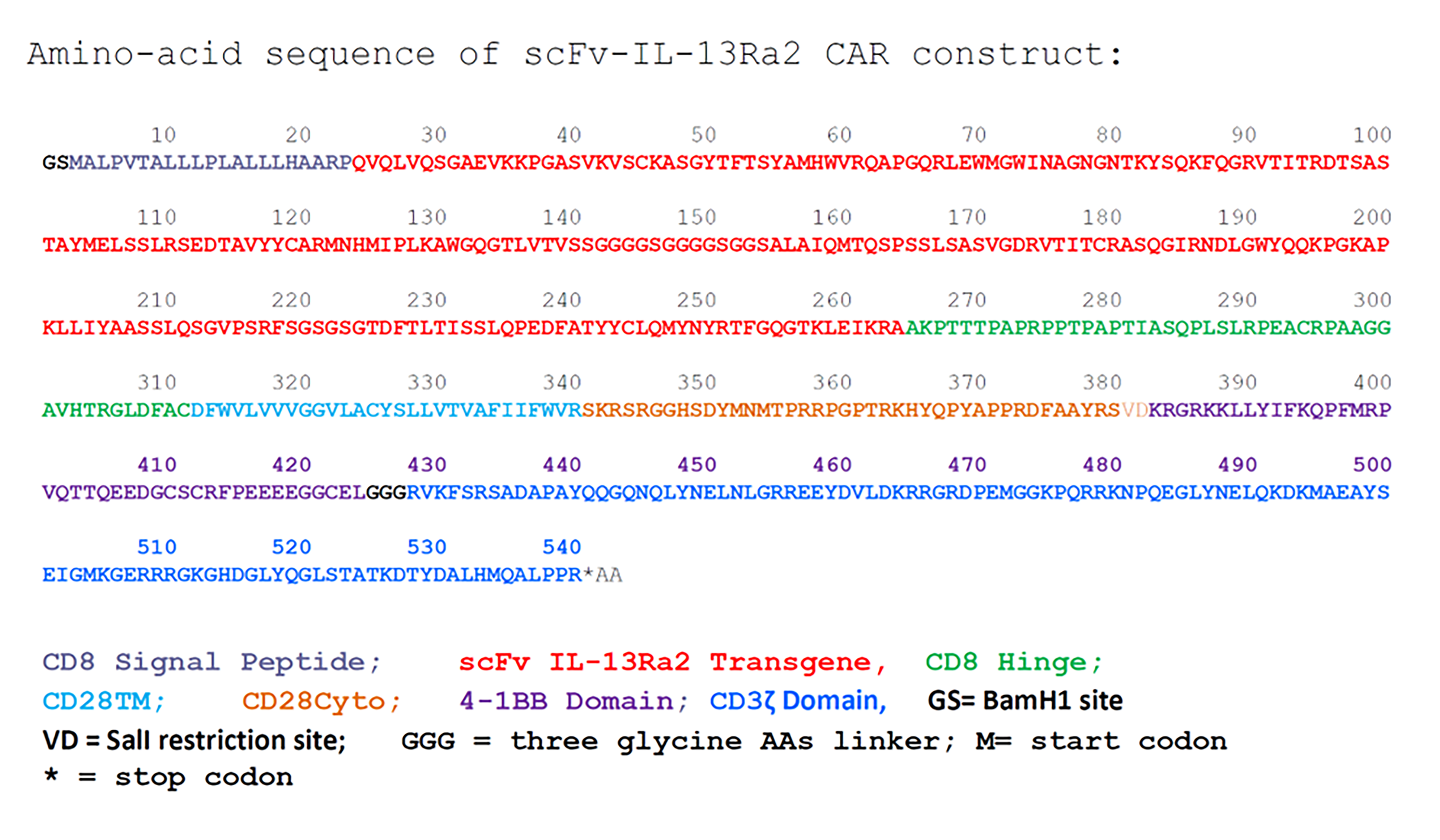

Supplement: Supplementary file 5 — (B). Amino acid sequence and its different domains. [file CTM2-14-e1664-s006.TIF]

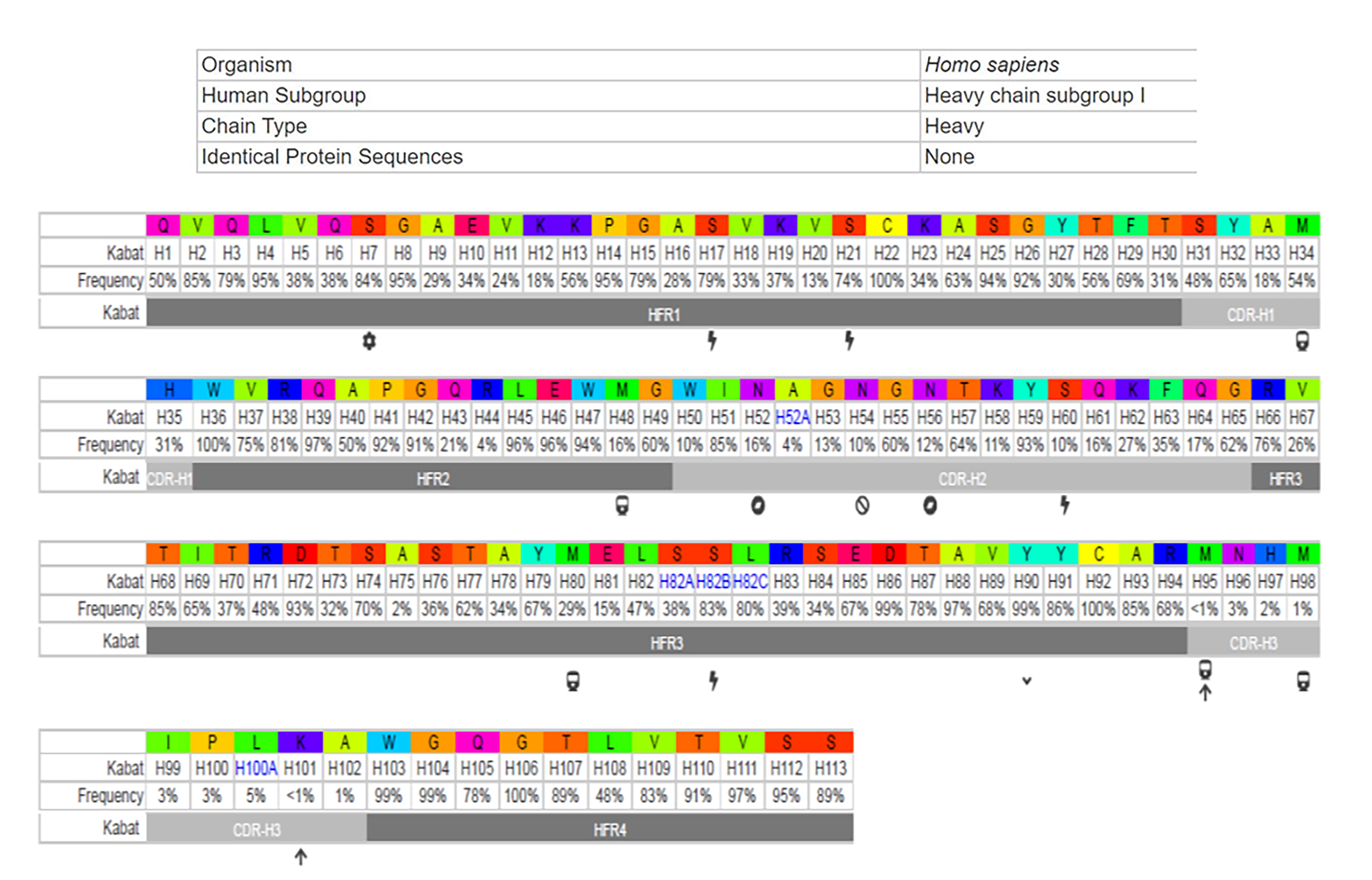

Supplement: Supplementary file 6 — Figure S3 (A) Amino acid numbering of heavy chain residues by Kabat numbering scheme. [file CTM2-14-e1664-s005.TIF]

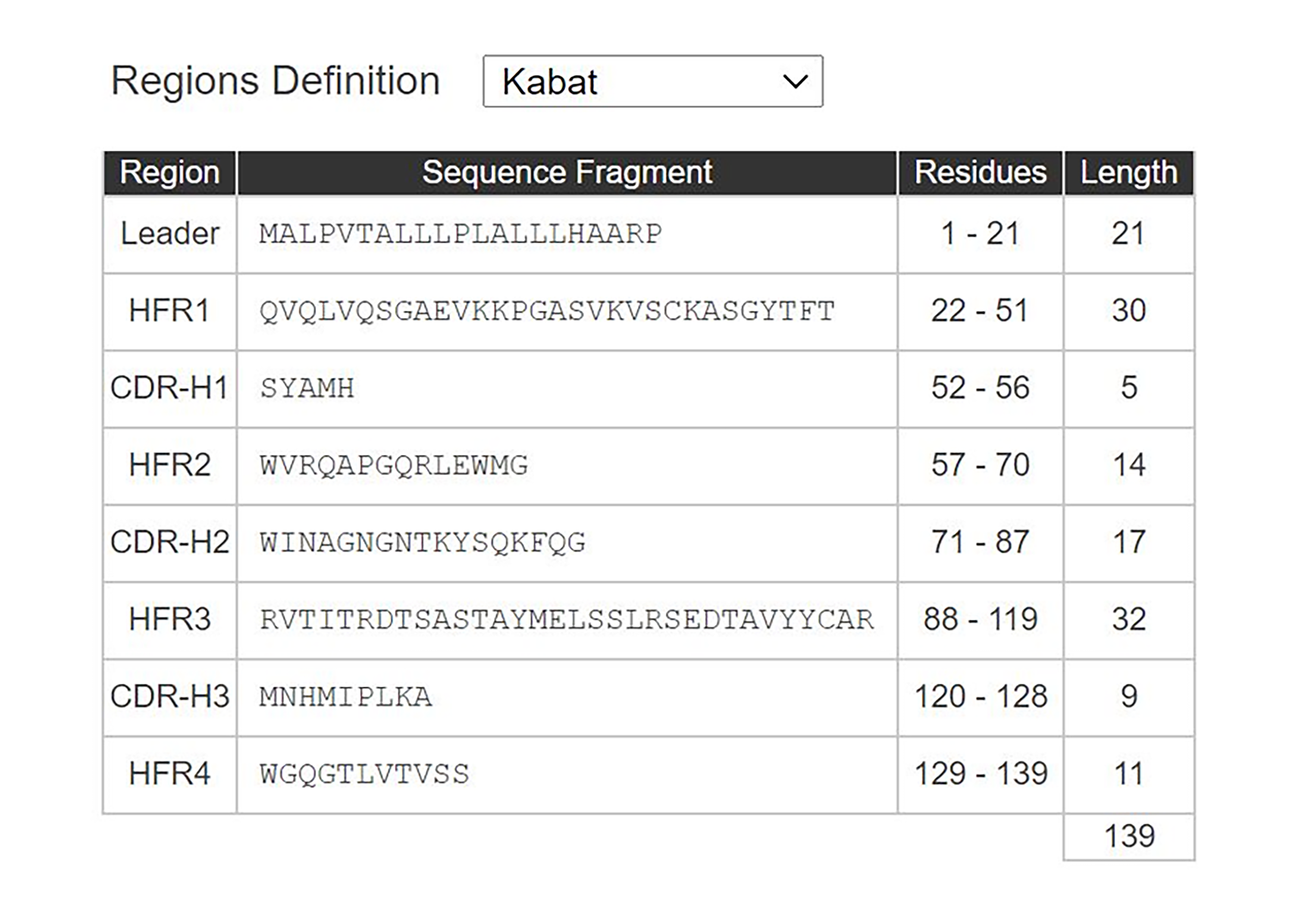

Supplement: Supplementary file 7 — (B) Analysis of heavy chain amino acids, their numbers and length in each region. [file CTM2-14-e1664-s011.TIF]

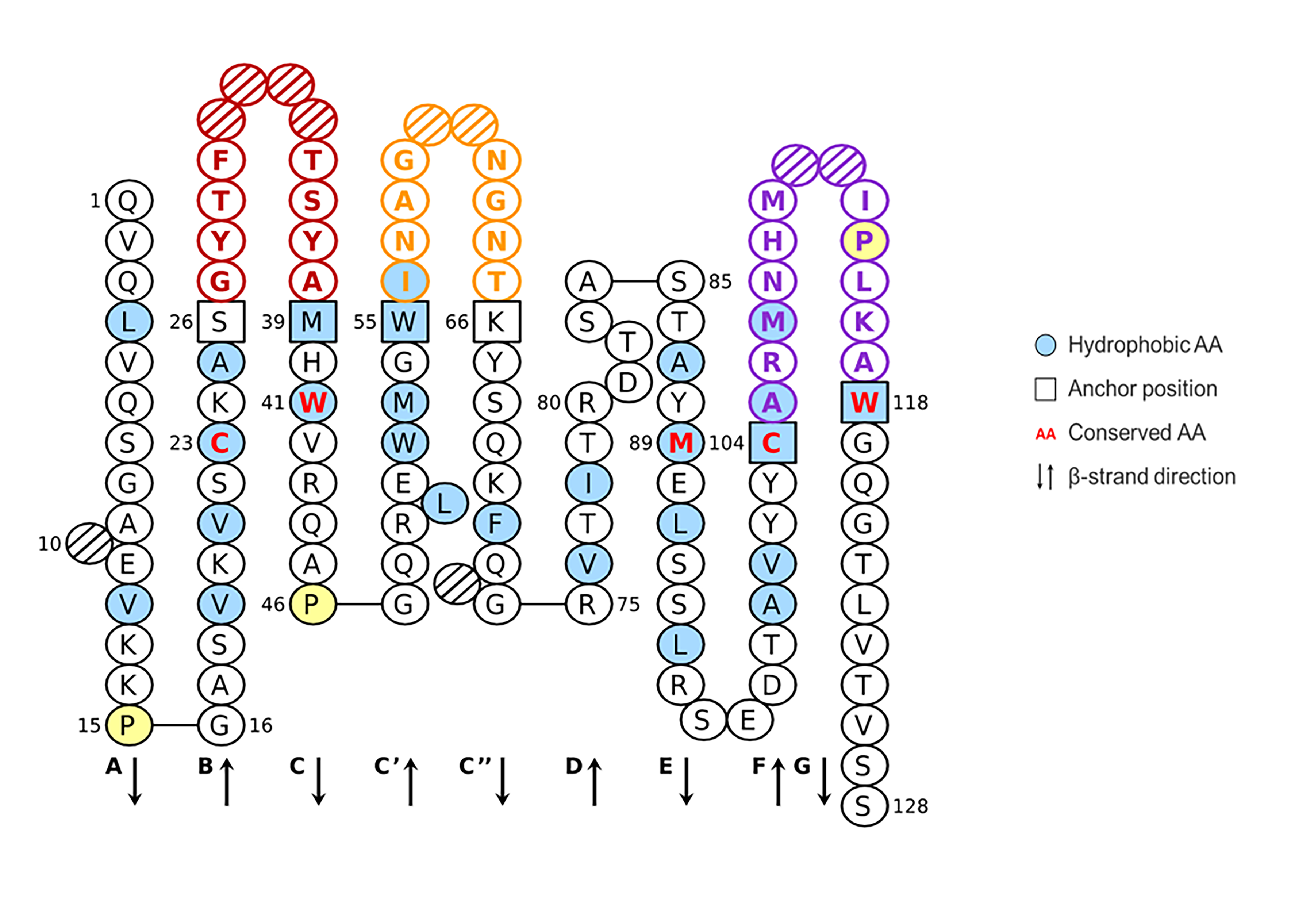

Supplement: Supplementary file 8 — (C) Loop structure of CDRs and amino acid residue position number—heavy chain: shown in blue circle are hydrophobic residues, anchor position, conserved amino acids AA, β‐strand direction. [file CTM2-14-e1664-s002.TIF]

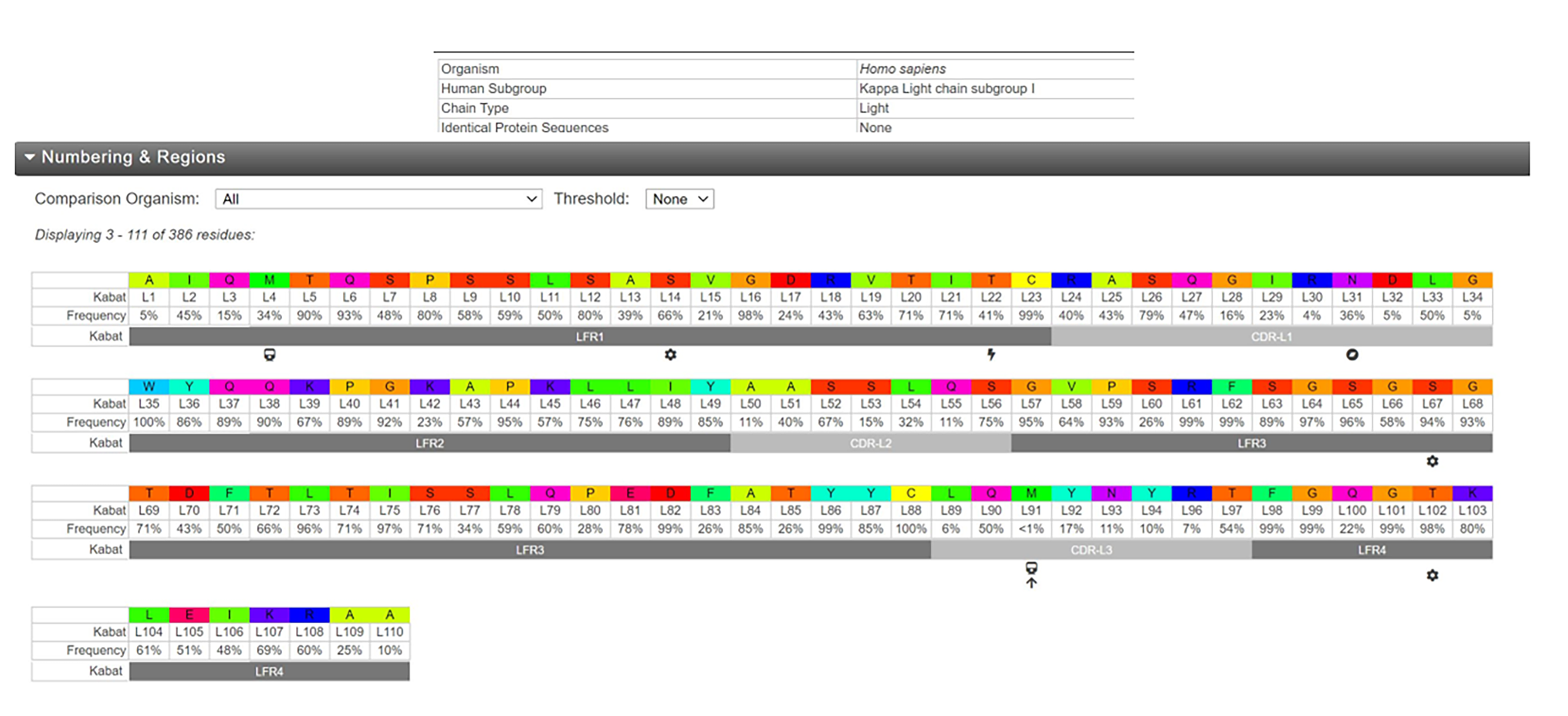

Supplement: Supplementary file 9 — Figure S4 (A) Amino acid numbering of Light chain residues with Kabat numbering scheme. [file CTM2-14-e1664-s012.TIF]

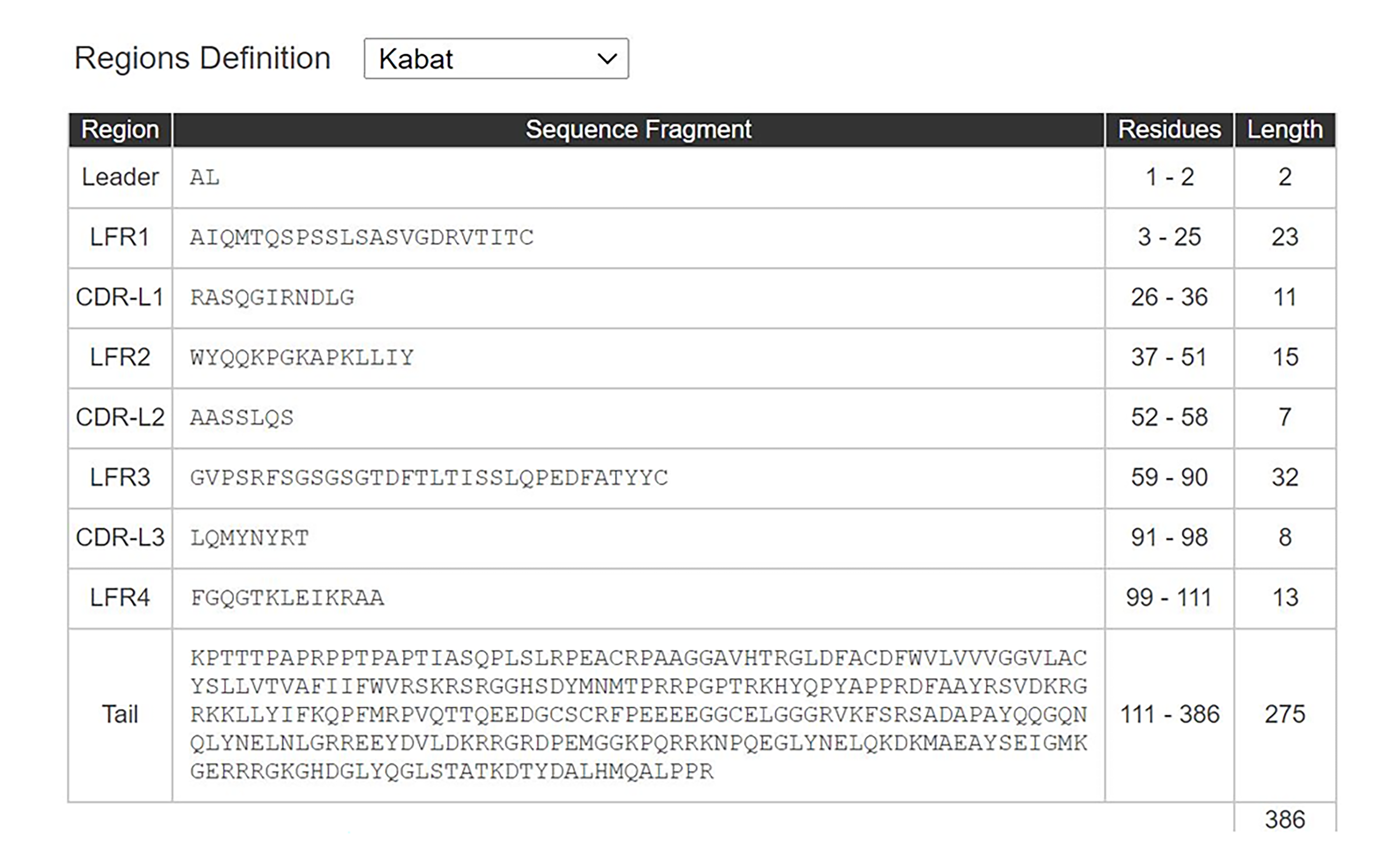

Supplement: Supplementary file 10 — (B) Analysis of light chain amino acids, their numbers and length in each region. [file CTM2-14-e1664-s003.TIF]

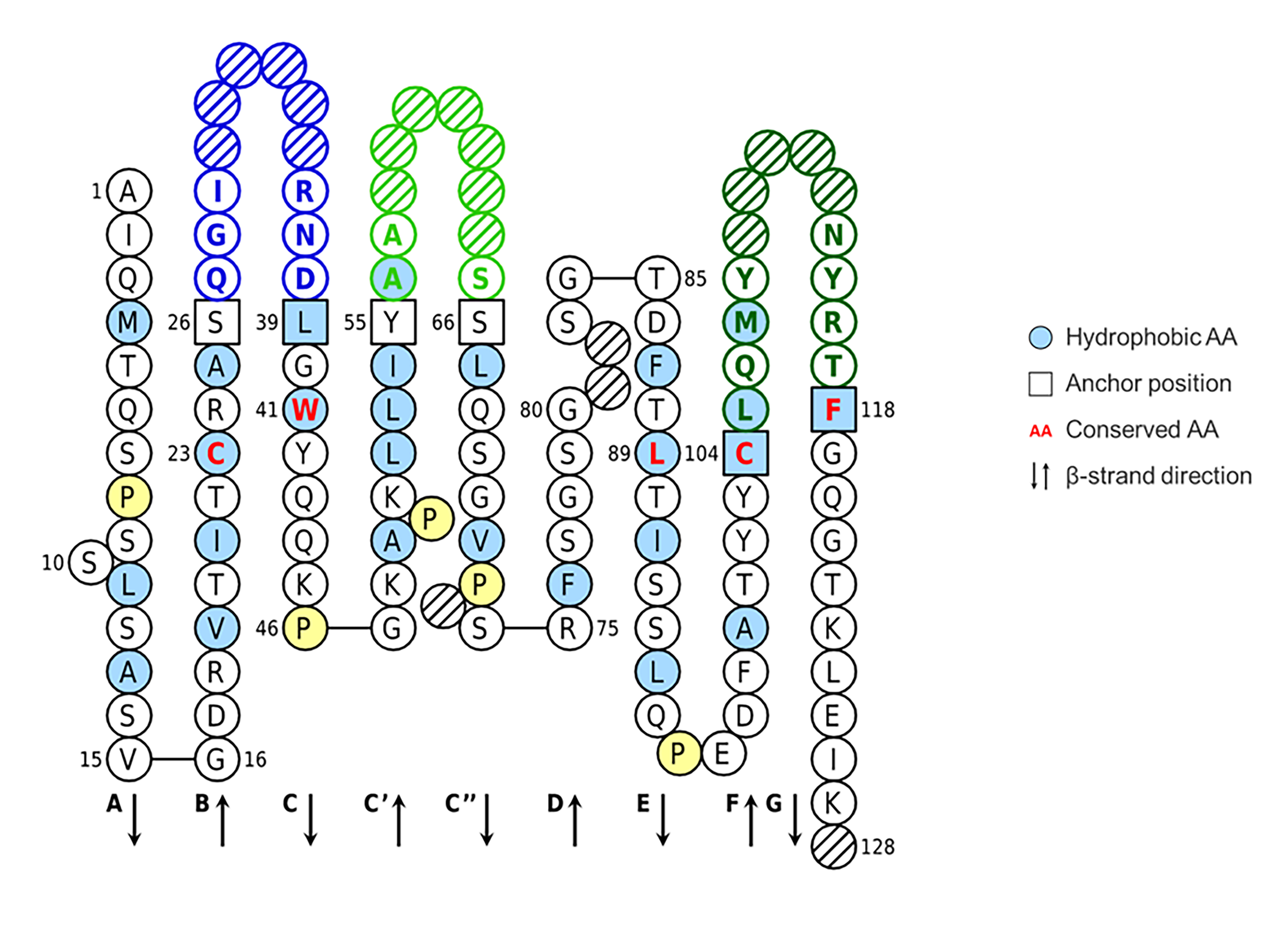

Supplement: Supplementary file 11 — (C) Loop structure of CDRs and amino acid residue position number—light chain: shown in blue circle are hydrophobic residues, anchor position, conserved amino acids AA, β‐strand direction. [file CTM2-14-e1664-s009.TIF]

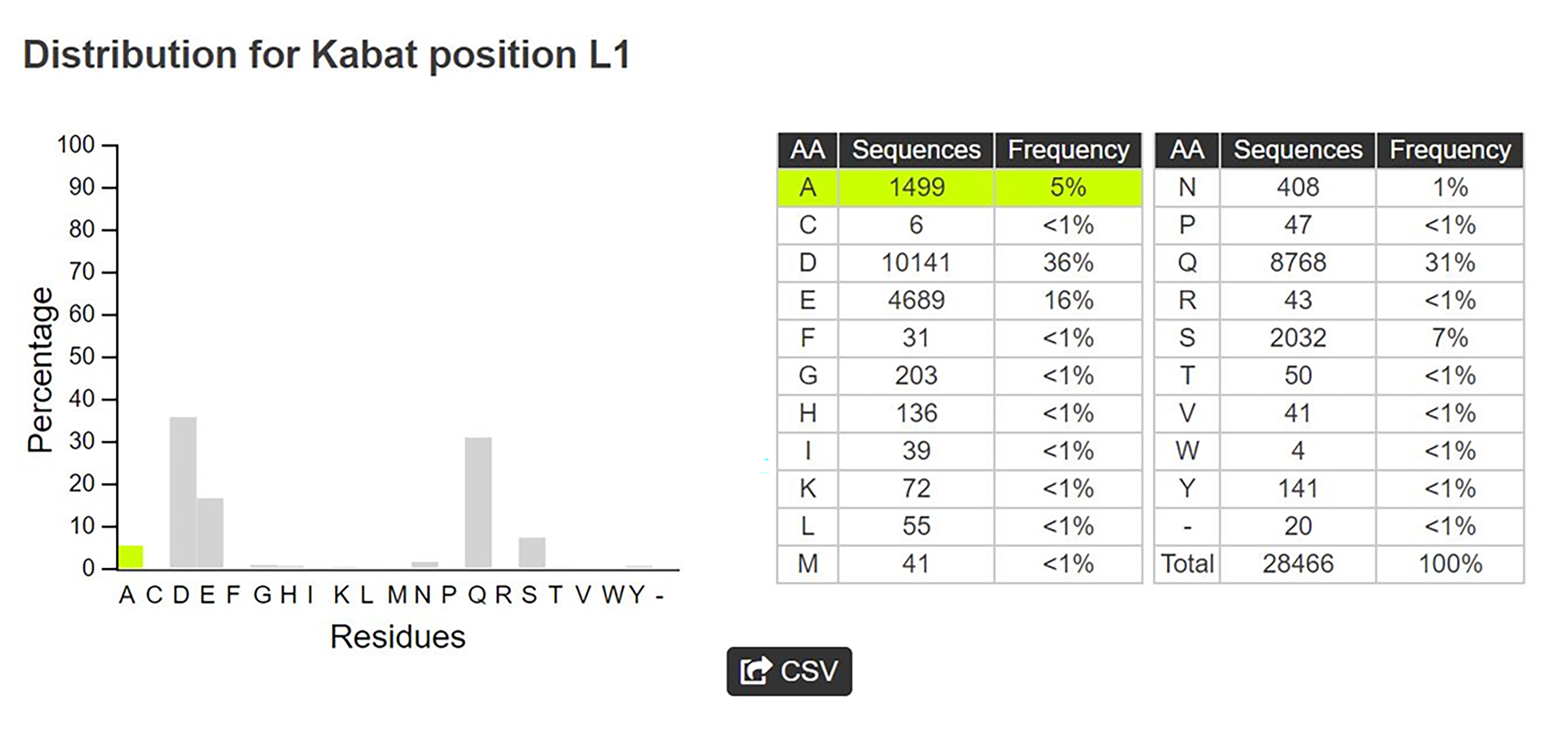

Supplement: Supplementary file 12 — Figure S5 (A) Distribution of heavy chain amino acids and their respective frequency. [file CTM2-14-e1664-s008.TIF]

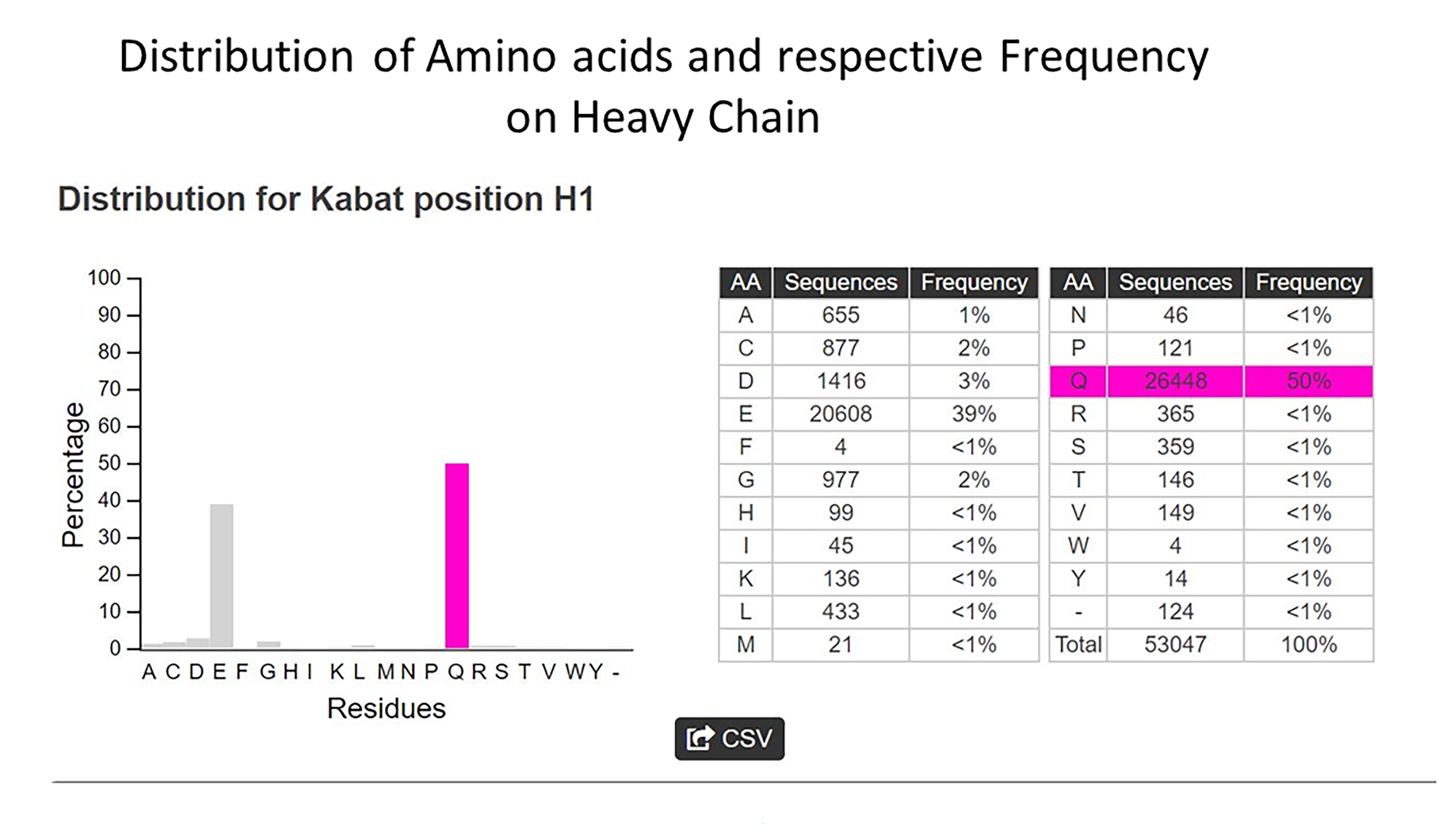

Supplement: Supplementary file 13 — (B) Distribution of light chain amino acids and their respective frequency. [file CTM2-14-e1664-s017.TIF]

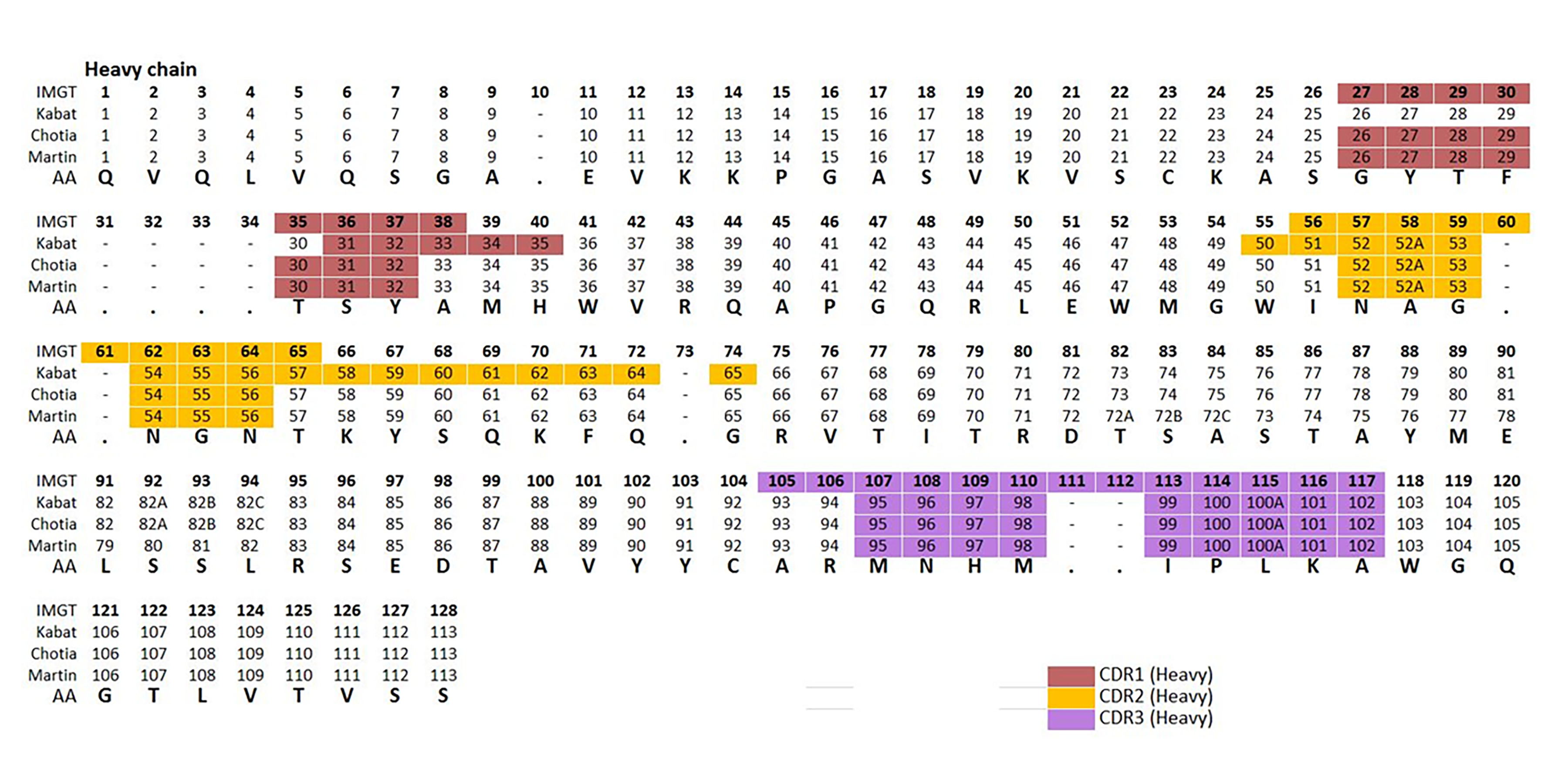

Supplement: Supplementary file 14 — Figure S6 (A) Numbering of scFv residue sequence for a comparative alignment analysis—heavy chain. Alignment of heavy chain residues according to IMGT, Kabat, Chothia and Martin numbering schemes. In red CDR1, orange CDR2 and purple CDR3 are shown. [file CTM2-14-e1664-s013.TIF]

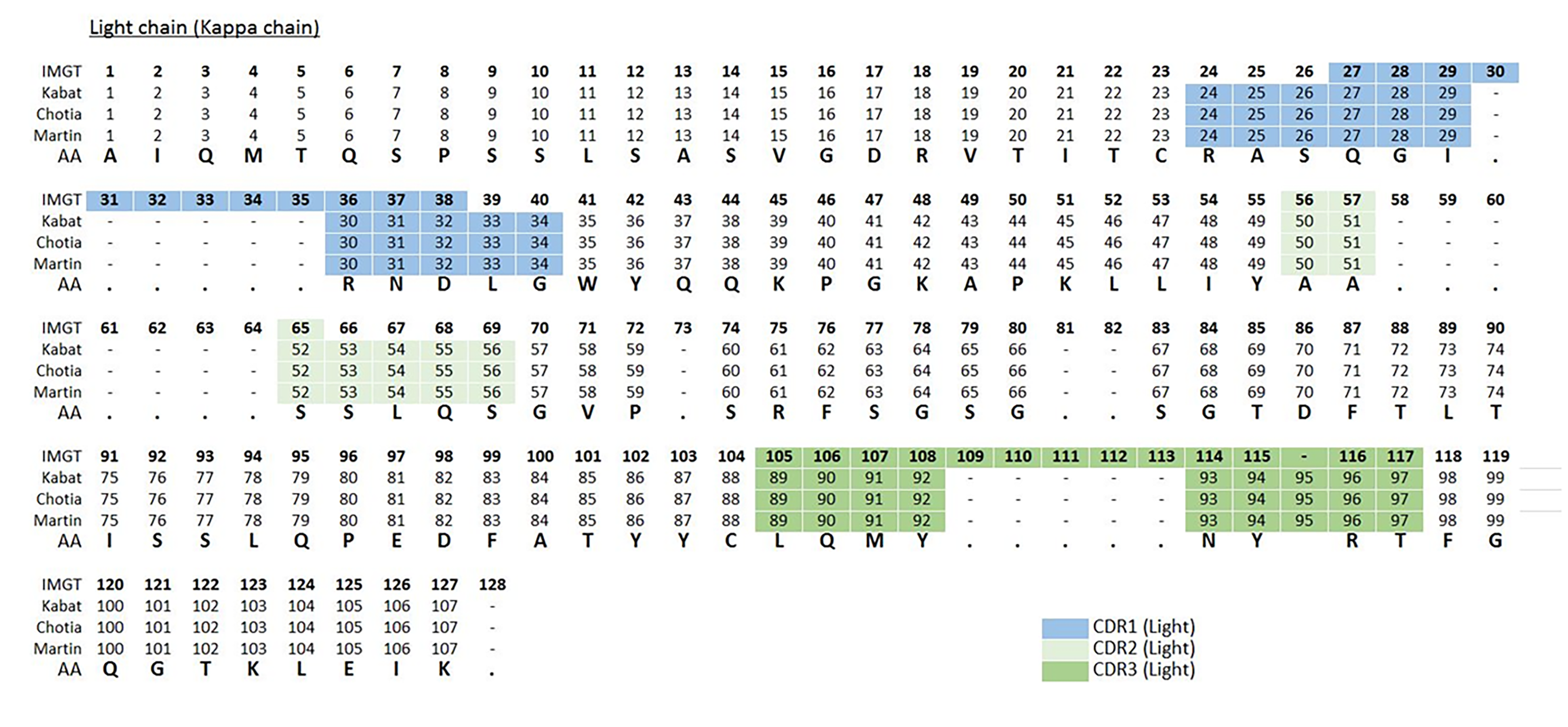

Supplement: Supplementary file 15 — (B) Numbering of scFv residue sequence for a comparative alignment analysis— light chain. Alignment of heavy chain residues according to IMGT, Kabat, Chothia and Martin numbering schemes. In blue CDR1, light green CDR2 and green CDR3 are shown. [file CTM2-14-e1664-s007.TIF]

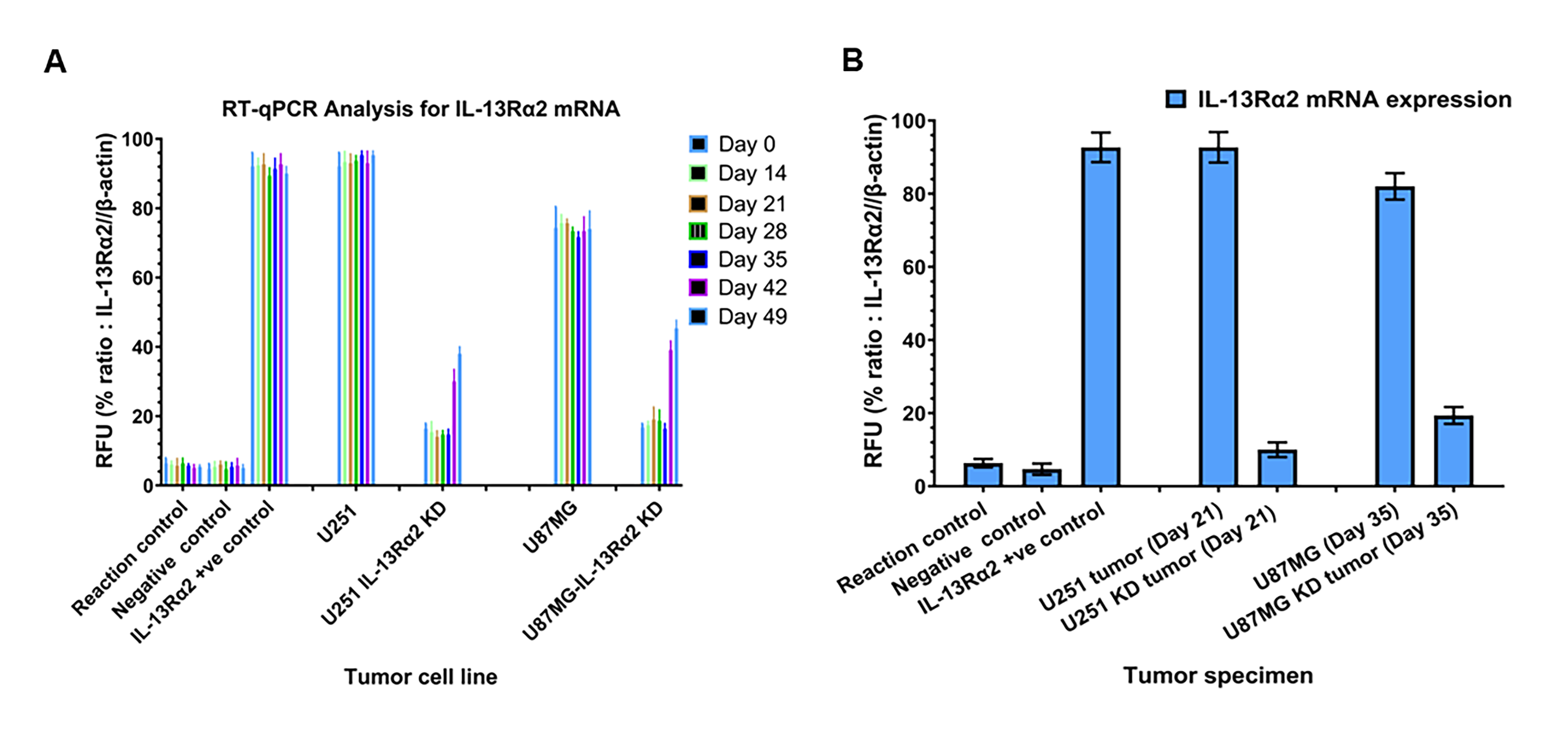

Supplement: Supplementary file 16 — Figure S7 (A) Analysis of RT‐qPCR analysis for IL‐13Rα2 RNA expression from U251 and U87MG glioma cell lines at different time points up to 49 days. Each value is a mean ± SD (standard deviation) of four independent experiments performed in triplicate. (B) IL‐13Rα2 gene silencing was examined by RT‐qPCR in U251 and U87MG xenograft tumour tissues at the end of the experiment. Each value is a mean ± SD of four independent experiments performed in triplicate. [file CTM2-14-e1664-s001.TIF]

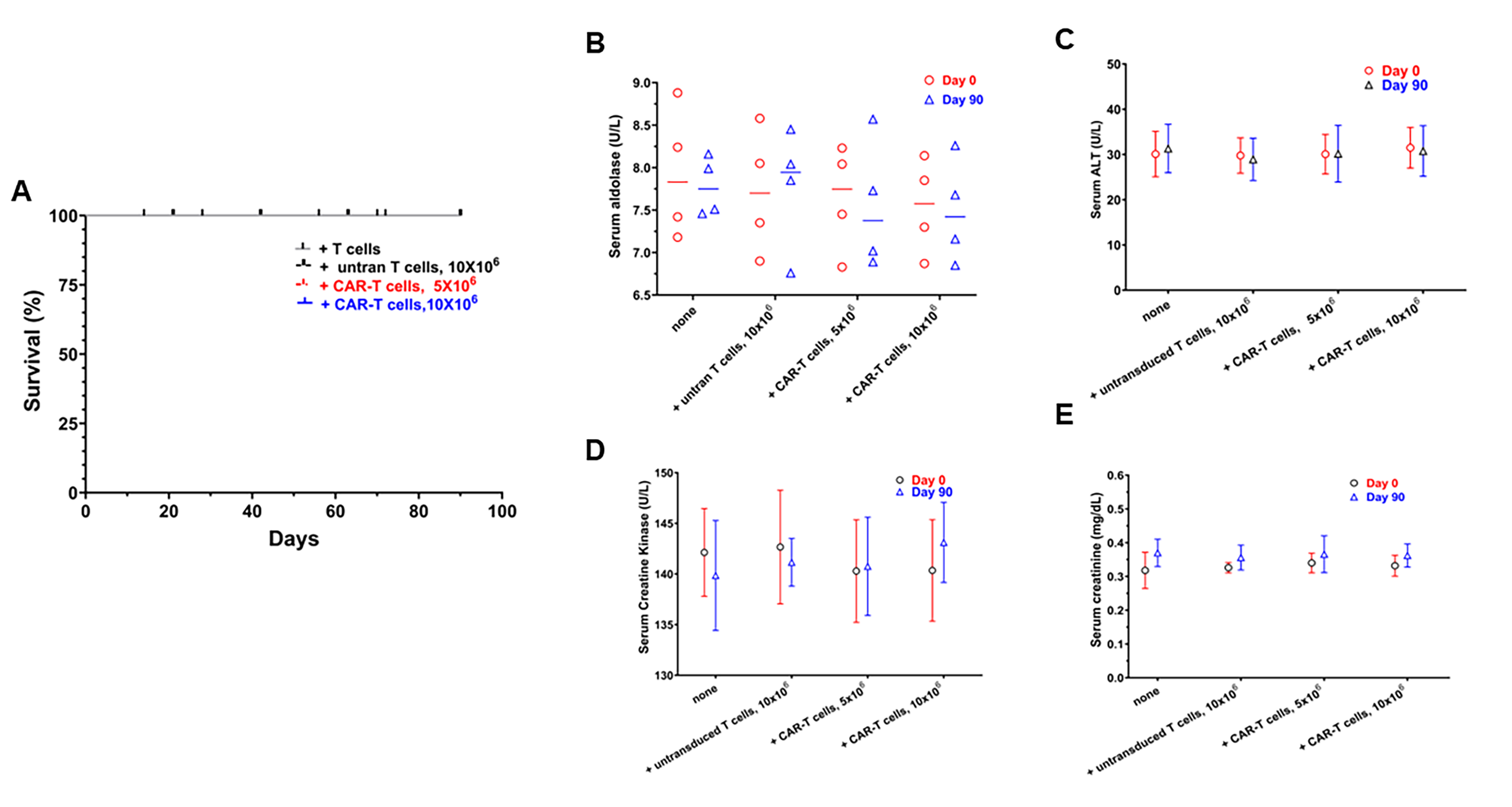

Supplement: Supplementary file 17 — Figure S8 (A) K‐M survival of non‐tumour bearing mice treated with untransduced and CAR‐T cells. Each datum is an average ± SD of two independent experiments each consisted of six mice: (B) serum chemistry analysis for the serum aldolase activity, (C) serum ALT activity, (D) serum CK activity, and (E) serum creatinine from mice sera collected on day 0 and 90. Each value represents mean ± SD of triplicate analysis of the samples from two independent experiments. [file CTM2-14-e1664-s004.TIF]
