# Supplementary material for: Identification and characterisation of novel CAR‐T cells to target IL13Rα2 positive human glioma in vitro and in vivo
Source: Clin Transl Med. 2024 Apr 29;14(5):e1664. doi: 10.1002/ctm2.1664 (PMC11058282; doi:10.1002/ctm2.1664)
Supplement: Supplementary file 18 — Supporting Information [file CTM2-14-e1664-s015.docx]

**Supplementary Table 1: Effect of CAR-T cell administration on Body Weight of non-tumor bearing Mice**

**Group Body Weight ***

**Day 0 Day 90**

**------------------ ---------------**

none 18.90 ± 1.56 19.20 ± 1.56

+ Untransduced T cells,10x10^6^ 20.05 ± 1.78 19.45 ± 1.78

+ CAR-T cells, 5x10^6^ cells 18.62 ± 1.48 19.62 ± 1.78

+ CAR-T cells, 10x10^6^ cells 19.04 ± 1.78 20.84 ± 1.68

__________________________________________________________________

*=each value represents mean ± SD gm body weight of mouse.
